# Repeated evolution of cytochrome P450-mediated spiroketal steroid biosynthesis in plants

**DOI:** 10.1038/s41467-019-11286-7

**Published:** 2019-07-19

**Authors:** Bastien Christ, Chengchao Xu, Menglong Xu, Fu-Shuang Li, Naoki Wada, Andrew J. Mitchell, Xiu-Lin Han, Meng-Liang Wen, Makoto Fujita, Jing-Ke Weng

**Affiliations:** 1 Whitehead Institute for Biomedical Research, 455 Main Street, Cambridge, MA 02142 USA; 2 Department of Applied Chemistry, School of Engineering, The University of Tokyo, 7-3-1 Hongo, Bunkyo-ku, Tokyo, 113-8656 Japan; 3 State Key Laboratory for Conservation and Utilization of Bio-Resources in Yunnan, Yunnan Institute of Microbiology, School of Life Sciences, Yunnan University, Kunming, Yunnan 650091 China; 4 Division of Advanced Molecular Science, Institute for Molecular Science, National Institutes of Natural Sciences, 5-1 Higashiyama, Myodaiji-cho, Okazaki, Aichi 444-8787 Japan; 5 Department of Biology, Massachusetts Institute of Technology, Cambridge, MA 02139 USA; 6 Present Address: Agroscope, Route des Eterpys 18, 1964 Conthey, Switzerland

**Keywords:** Enzymes, Plant evolution, Biocatalysis

## Abstract

Diosgenin is a spiroketal steroidal natural product extracted from plants and used as the single most important precursor for the world steroid hormone industry. The sporadic occurrences of diosgenin in distantly related plants imply possible independent biosynthetic origins. The characteristic 5,6-spiroketal moiety in diosgenin is reminiscent of the spiroketal moiety present in anthelmintic avermectins isolated from actinomycete bacteria. How plants gained the ability to biosynthesize spiroketal natural products is unknown. Here, we report the diosgenin-biosynthetic pathways in himalayan paris (*Paris polyphylla*), a monocot medicinal plant with hemostatic and antibacterial properties, and fenugreek (*Trigonella foenum–graecum*), an eudicot culinary herb plant commonly used as a galactagogue. Both plants have independently recruited pairs of cytochromes P450 that catalyze oxidative 5,6-spiroketalization of cholesterol to produce diosgenin, with evolutionary progenitors traced to conserved phytohormone metabolism. This study paves the way for engineering the production of diosgenin and derived analogs in heterologous hosts.

## Abstract (web summary)

Diosgenin is a spiroketal natural product that is used as a precursor in the industrial synthesis of steroids. Here, the authors identified key cytochrome P450 enzymes responsible for the conversion of cholesterol to diosgenin from two phylogenetical distinct diosgenin-producing plant species.

## Introduction

Sterols are isoprene-derived tetracyclic triterpenoid lipids that play essential roles in modulating membrane fluidity, intracellular transport, and cell signaling in all eukaryotes^1–3^. Sterols are also the precursors of a plethora of specialized metabolites, such as steroid hormones, bile acids, steroidal saponins, and steroidal glycoalkaloids, which serve diverse functions ranging from defense to regulation of specific aspects of physiology in response to environmental cue^4,5^. The chemical diversity of specialized steroids can be largely attributed to the burgeoning catalytic activities from a number of enzyme families, including but not limited to 2,3-oxidosqualene cyclases, cytochromes P450 (CYPs), oxidoreductases, and UDP-dependent glycosyltransferases^6,7^. The emergence and subsequent diversification of specialized sterol metabolic systems driven by the multifactorial selection pressures imposed by the ever-changing biotic and abiotic environments have shaped eukaryotic evolution^8^.

**Fig. 1:**
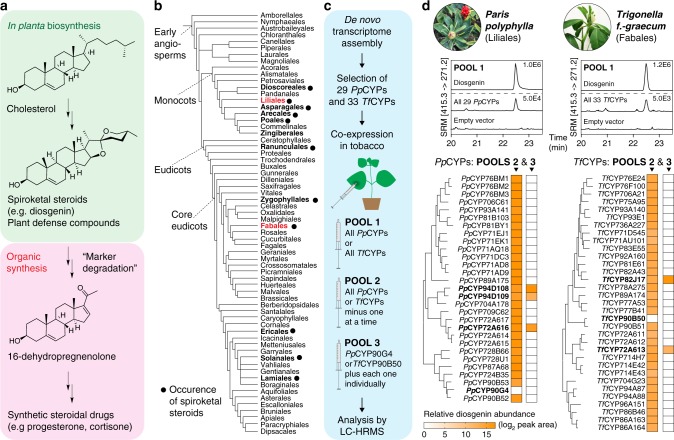
Identification of diosgenin-biosynthetic cytochrome P450s using pooled-screen approach. **a** Diosgenin is biosynthetically derived from cholesterol, and used as the precursor for the synthetic production of steroidal drugs via “Marker degradation”^14^. **b** Phylogenetic relationship of plant families that reportedly harbor spiroketal steroid chemotypes. *P. polyphylla* and the *T. fenum–graecum*, belonging to Liliales and Fabales respectively, are the two species investigated in this study. Angiosperm phylogeny was obtained from Theodor C.H. Cole, Hartmut H. Hilger, and Peter F. Stevens. **c** The stepwise pooled-screen approach employed in this study to identity diosgenin-biosynthetic *CYP*s from *P. polyphylla* and *T. fenum–graecum*. The screens were conducted using the combinatorial transient gene expression system in *Nicotiana benthamiana*. **d** Identification of the diosgenin-biosynthetic *CYP*s from *P. polyphylla* and *T. fenum–graecum*. 29 *CYP*s from *P. polyphylla* (*PpCYP*s) and 33 *CYP*s from *T. foenum–graecum* (*TfCYP*s) were selected for the screens. In the initial screen (POOL 1), co-expression of all *PpCYP*s or all *TfCYP*s resulted in diosgenin accumulation in *N. benthamiana*, shown in selected reaction monitoring chromatograms (SRM) from LC–MS data (peak intensity is indicated). In the second step (POOL 2), batches of *CYP*s omitting one *CYP* at a time were tested. *PpCYP90G4* and *TfCYP90B50* were identified as essential for reconstituting diosgenin production in *N. benthamiana*. In POOL 3, each *PpCYP* or *TfCYP* was co-expressed with *PpCYP90G4* or *TfCYP90B50*, respectively. Three *PpCYP*s (*PpCYP94D108*, *PpCYP94D109,* and *PpCYP72A616*) and two *TfCYP*s (*TfCYP82J17* and *TfCYP72A613*) were identified as capable of completing diosgenin biosynthesis together with *PpCYP90G4* or *TfCYP90B50*. CYP candidates are organized based on their maximum-likelihood phylogenetic relationship. The relative abundance of diosgenin accumulation in transgenic *N. benthamiana* is shown as heat map

Diosgenin is the aglycone of a class of spirostanol-type saponins sporadically distributed in numerous monocot and eudicot plant species, which are important defense compounds with a multitude of antimicrobial and antiherbivory activities (Fig. 1a, b)^9–11^. For instance, diosgenin is a major natural product of *Paris polyphylla* (a monocot) and *Trigonella foenum–graecum* (a eudicot), two plants with rich histories used by humans for medicinal and/or culinary purposes^12,13^. Diosgenin holds particular significance for the modern pharmaceutical industry. Extracted in large quantities from Mexican yam (*Dioscorea mexicana*), diosgenin is used as the main precursor for the synthesis of most steroidal drugs, including hormonal contraceptives and corticosteroid anti-inflammatory agents, through the “Marker degradation” process developed by American chemist Russell Earl Marker in the 1940s (Fig. 1a)^14^. The characteristic 5,6-spiroketal moiety in diosgenin is reminiscent of the 5,6- and 6,6-spiroketal moieties present in several polyketides isolated from soil bacteria^15^. In those systems, the spiroketal is formed via spontaneous cyclization of an epoxide-ketone or a dihydroxy-ketone intermediate, the latter of which is guided by a unique cyclase to ensure production of a single stereoisomer^15,16^. Considering these bacteria-specific enzymes have no known homologs in plants and an epoxide-ketone like mechanism would produce a hydroxylated diosgenin, it is likely that plants have developed an alternative yet unknown route to such spiroketalization.

Here, we report the elucidation of diosgenin biosynthesis from the common precursor cholesterol in *P. polyphylla* and *T. foenum–graecum*. Our results demonstrate that both species employ independently evolved CYPs to catalyze oxidative 5,6-spiroketalization of cholesterol to yield diosgenin. This work uncovers unknown enzymatic mechanisms underlying spiroketal steroid biosynthesis in plants, and paves the way for metabolic engineering of diosgenin production in alternative microbial or plant hosts.

## Results

### CYPs are involved in diosgenin biosynthesis

To delineate diosgenin biosynthesis through a candidate-gene approach^17^, we first sequenced and de novo assembled several tissue-specific transcriptomes of *P. polyphylla* (root, stem, leaf, and fruit) and *T. foenum–graecum* (stem, leaf, flower, and developing seed pods). As several eukaryotic CYPs have been reported to functionalize the hydrocarbon tail of sterols^18^, our initial hypothesis was that the spiroketal formation in diosgenin biosynthesis could potentially be mediated by one or more CYPs starting from cholesterol as the precursor, although other types of oxygenases may also be involved^19^. All CYP-encoding genes were hence identified from the de novo transcriptomes and manually annotated based on phylogenetic analyses and CYP nomenclature (Supplementary Figs. 1, 2). However, after excluding a number of highly conserved CYPs with well characterized functions, the list of candidate CYPs for diosgenin biosynthesis could not be narrowed down further owing to the difficulty of predicting CYP function based on sequence. With a set of simple assumptions, we devised a stepwise scheme of pooled screens to test CYP functions using the *Agrobacterium tumefaciens*-mediated transient gene expression system in *Nicotiana benthamiana* (Fig. 1c)^20^. Combinatorial expression of biosynthetic enzyme-encoding genes in *N*. *benthamiana* is a powerful platform for characterizing gene functions and large-scale metabolite and recombinant protein production^21^. For instance, such method enabled the elucidation of podophyllotoxin biosynthesis in *Podophyllum hexandrum*^22^. Furthermore, *N. benthamiana* accumulates substantial amount of cholesterol (∼ 12% of total sterols)^23^, and thus is well suited for screening candidate enzymes for diosgenin biosynthesis. We reasoned that if the minimum set of diosgenin-biosynthetic *CYP*s were contained within a larger test gene pool, co-expression of this pool of genes in *N. benthamiana* would likely result in diosgenin production, and the specific *CYP*s could be subsequently identified. We therefore selected 29 full-length *P. polyphylla CYP*s (*PpCYP*s) and 33 full-length *T. foenum–graecum CYP*s (*TfCYP*s), cloned each gene into the pEAQ-HT expression vector^20^, and individually transformed the resultant constructs into *A. tumefaciens* (Fig. 1c and Supplementary Dataset 1). For the initial pooled screen, *A. tumefaciens* strains containing each of the 29 *PpCYP* constructs or the 33 *TfCYP* constructs were cultured individually and mixed in equal volume prior to co-infiltration into *N. benthamiana* leaves (Fig. 1c: POOL 1). Metabolic profiling of the leaf extracts by liquid chromatography-mass spectrometry (LC–MS) analysis revealed that transient expression of both the *Pp*CYP and the *Tf*CYP pools indeed resulted in heterologous production of diosgenin in *N. benthamiana* (Fig. 1d: POOL 1). This result suggests that the diosgenin-biosynthetic *CYP*s from the two species were likely contained within each pool.

To identify the specific *CYP*s responsible for diosgenin synthesis within each pool, we next tested the essentiality of each *CYP* by withdrawing one *CYP* at a time from the pool (Fig. 1c: POOL 2). Interestingly, two CYPs of the CYP90 family, namely *Pp*CYP90G4 and *Tf*CYP90B50, were found to be indispensable for diosgenin production within their respective gene pools (Fig. 1d: POOL 2). Moreover, expression of *PpCYP90G4* or *TfCYP90B50* alone was not sufficient to produce diosgenin in *N. benthamiana*, suggesting that additional *CYP*s were yet necessary (Supplementary Fig. 3). In the third step, we co-expressed *PpCYP90G4* or *TfCYP90B50* with every other *CYP* from the respective gene pool (Fig. 1c, d: POOL 3). This experiment revealed that *PpCYP90G4* in combination with *PpCYP94D108*, *PpCYP94D109* or *PpCYP72A616*, and *TfCYP90B50* in combination with *TfCYP72A613* or *TfCYP82J17* were sufficient to elicit diosgenin production in *N. benthamiana*. Together, our pooled-screen approach successfully identified several pairs of *CYP*s from *P. polyphylla* and *T. foenum–graecum* that are both necessary and sufficient to reconstitute diosgenin biosynthesis in *N. benthamiana*.

### CYP pairs catalyze 5,6-spiroketalization of cholesterol

**Fig. 2:**
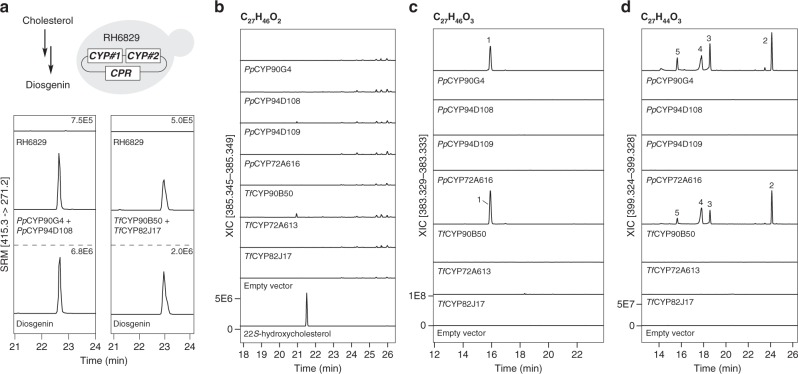
Elucidation of CYP-mediated diosgenin biosynthesis from cholesterol. **a** Reconstitution of diosgenin biosynthesis in the yeast strain RH6829, which was engineered to accumulate cholesterol^25^. Co-expression of *CYP* pairs *PpCYP90G4*/*PpCYP94D108* or *TfCYP90B50*/*TfCYP82J17* together with a *CYP reductase* (*CPR*) from *A. thaliana* (*ATR1*) led to diosgenin production in RH6829 yeast, shown in selected reaction monitoring chromatograms (SRM) from LC–MS data (peak intensity is indicated). **b**–**d** LC-HRMS-based metabolic profiling of transgenic *N. benthamiana* expressing individual diosgenin-biosynthetic *CYP*s from *P. polyphylla* and *T. foenum–graecum*. Extracted ion chromatograms (XICs) corresponding to putative monohydroxylated cholesterol with predicted molecular formula of C_27_H_46_O_2_ (identified as [M + H-H_2_O]^+^ ions) **b**, and putative dihydroxylated-cholesterol derivatives C_27_H_46_O_3_ (identified as [M + H-2H_2_O]^+^) **c** and C_27_H_44_O_3_ (identified as [M + H-H_2_O]^+^ ions) **d** are displayed. Major products detected in *N. benthamiana* expressing *PpCYP90G4* or *TfCYP90B50* are labeled as compounds **1**–**5**

To exclude the possibility that some endogenous *N. benthamiana* enzymes might be involved in diosgenin synthesis from cholesterol in addition to the identified *CYP* pairs as described above, we sought to reconstitute diosgenin biosynthesis in yeast. Two *CYP* pairs, *Pp*C*YP90G4*-*PpCYP94D108,* and *TfCYP90B50*-*TfCYP82J17*, were selected for this experiment because they yielded more diosgenin in *N. benthamiana* than the other *CYP* pairs from the same host species (Supplementary Fig. 4). Each *CYP* pair was co-expressed together with an Arabidopsis *CYP reductase*^24^ in the yeast strain RH6829, which was engineered to accumulate cholesterol (Fig. 2a)^25^. Similar to the *N. benthamiana* experiment, transgenic expression of either *Pp*C*YP90G4*-*PpCYP94D108* or *TfCYP90B50*-*TfCYP82J17* pair resulted in diosgenin production in RH6829 yeast (Fig. 2a). These results further support that these specific CYP pairs are sufficient to reconstitute diosgenin biosynthesis from cholesterol in heterologous hosts.

**Fig. 3:**
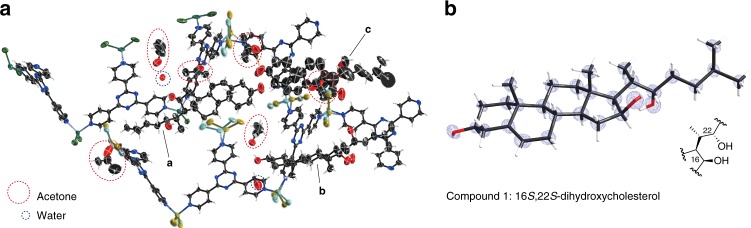
Absolute structural elucidation of 1 by crystalline sponge X-ray diffraction analysis. **a** ORTEP diagram with 50% probability showing the asymmetric unit (ASU) of crystalline sponge-compound **1** complex. A single crystal of [(ZnCl_2_)_3_(tpt)_2_]_*n*_ [tpt = 2,4,6-tri(4-pyridyl)-1,3,5-triazine] was used as the crystalline sponge. Three crystallographically independent molecules of **1** were observed in the ASU **a**–**c**. The solvent molecules acetone and water are highlighted by red and blue circles, respectively. **b** The structural model of **1** overlaid on the electron density map *F*_o_ contoured at the 2.0σ level (2.6 eÅ^−3^). The absolute stereochemistry of C16 and C22 is highlighted

To probe into the detailed biochemical mechanisms underlying CYP-mediated diosgenin biosynthesis, we assessed the enzymatic activity of each CYP individually. To our surprise, none of the CYPs identified in our screen seemed to catalyze the canonical monohydroxylation reaction with cholesterol as the substrate when expressed in *N. benthamiana* (Fig. 2b). Nevertheless, expressing *PpCYP90G4* or *TfCYP90B50* alone in *N. benthamiana* produced nearly identical profiles of reaction products with mass-to-charge (m/z) ratios corresponding to polyoxygenated cholesterols (compounds **1**–**5**, Fig. 2b–d). Compound **1** has a predicted chemical formula of C_27_H_46_O_3_, suggesting a dihydroxylated cholesterol. Expressing *PpCYP90G4* or *TfCYP90B50* alone in yeast also consistently produced **1** (Supplementary Fig. 5). We therefore purified 1.5 mg of **1** from yeast cells expressing *PpCYP90G4*, and determined its absolute structure using the recently developed NMR-coupled crystalline sponge X-ray diffraction analysis^26,27^. The absolute structure of **1** could be unequivocally resolved as 16*S*,22*S*-dihydroxycholesterol by X-ray diffraction analysis of the crystalline sponge-compound 1 complex alone (Fig. 3, Supplementary Fig. 6 and Supplementary Table 1). The positions of the two hydroxyl groups at C16 and C22 were independently confirmed by ^1^H, ^13^C, DEPT, HSQC, and HMBC NMR analyses, whereas their relative configurations were further supported by additional NOESY NMR analysis (Supplementary Figs. 7–11 and Supplementary Table 2). The absolute structural elucidation of **1** revealed that *Pp*CYP90G4 and *Tf*CYP90B50 are capable of catalyzing stereospecific 16- and 22-dihydroxylation of cholesterol to yield 16*S*,22*S*-dihydroxycholesterol as one of the main enzymatic products.

Compounds **2**–**5** were unstable during our attempted purification process, precluding direct structural elucidation. We therefore inferred their structures indirectly based on various sources of information including MS analysis, retention time, compound degradation behaviors, known CYP-catalyzed chemistry and chemical logic. Compounds **2**–**5** all share the predicted chemical formula of C_27_H_44_O_3_, together with characteristic MS^2^ fragmentation patterns^28^, suggesting two categories of isomeric products (Supplementary Figs. 12–14). Compounds **3** and **5** are 16-oxo-22-hydroxy-cholesterol and 16-hydroxy-22-oxo-cholesterol respectively, likely derived from **1** upon additional *Pp*CYP90G4 or *Tf*CYP90B50-dependent hydroxylation at C16 or C22 followed by dehydration (Supplementary Figs. 12–15). Compounds **2** and **4** share nearly identical MS^2^ fragmentation patterns featuring two previously reported signature daughter ions (m/z of 253.195 and 271.206) indicative of furostanol-type steroids (Supplementary Figs. 12, 13)^28^. We therefore concluded that **2** and **4** are two furostanol diastereomers resulting from furoketalization of **5** (Supplementary Figs. 12–15). Compound **3** theoretically could also cyclize to produce the corresponding hemiketals (Supplementary Fig. 15), but these products were not detected in appreciable amounts in both the *N. benthamiana* and yeast experiments. Compounds **1**, **2**, and **4** could also be detected in various tissues of *P. polyphylla* and *T. foenum–graecum*, confirming the accumulation of these key intermediates in planta (Supplementary Fig. 16).

**Fig. 4:**
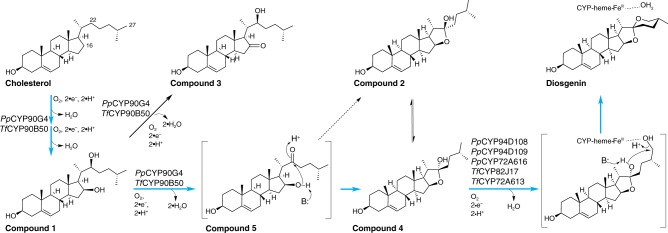
The proposed catalytic sequence of CYP-mediated diosgenin biosynthesis from cholesterol. The on-pathway catalytic steps are colored in blue. The identity of the key intermediates were determined directly or inferred indirectly. Other possible mechanisms for the second cyclization step are presented in Supplementary Fig. 20

To further elucidate the enzymatic and non-enzymatic processes that give rise to these polyoxygenated cholesterol products and identify the true diosgenin-biosynthetic intermediates, we performed in vitro enzyme assays using microsomes prepared from RH6829 yeast expressing individual *CYP*s together with the Arabidopsis *CYP reductase* (Supplementary methods and Supplementary Fig. 17)^24^. We found that the cholesterol substrate produced by the RH6829 yeast as well as steroids produced by certain CYPs during yeast growth could be co-purified with the microsome. This circumvents the need of adding steroidal substrates of extremely low water solubility to the aqueous assay buffer, which was intractable in our hands. Compounds **1** and **4** were present as the two main enzymatic products of *Pp*CYP90G4 when mixing microsomes prepared from the control RH6829 yeast and yeast expressing *PpCYP90G4*. Furthermore, mixing microsomes from yeast strains expressing *PpCYP90G4* and *PpCYP94D108* respectively resulted in time-dependent de novo production of diosgenin at the expense of **4**. Of note, we observed that **4** dissolved in solvent devoid of enzyme gradually isomerizes to give **2**, and eventually reaches equilibrium with **4** over the course of several days (Supplementary Fig. 18). Moreover, **2** was present only in trace amount at the beginning of the assay, indicating that it is unlikely an immediate enzymatic product of *Pp*CYP90G4 and the initial furoketalization likely occurs within the *Pp*CYP90G4 active site in a stereospecific manner (Supplementary Fig. 15). Altogether, these results suggest that **1**, **4**, and **5** are true diosgenin-biosynthetic intermediates, whereas some of the other polyoxygenated products observed in our in vivo reconstitution experiments (i.e., **2** and **3**) are likely off-pathway side products resulted from the intrinsic reactivity of the involved CYPs and their enzymatic products (Fig. 4). Our data also indicate that the third catalytic cycle of cholesterol oxidation (converting **1**–**4)** mediated by *Pp*CYP90G4 or *Tf*CYP90B50 is likely the rate-limiting step of the whole biochemical sequence. It is worth mentioning that oxidative ring closure bridging C16 and C22 in diosgenin biosynthesis is catalyzed by a single CYP90 in both *P. polyphylla* and *T. foenum–graecum*, which is dissimilar with the analogous reaction catalyzed by an iron/2-oxoglutarate-dependent oxygenase (GAME11) in steroidal alkaloid biosynthesis in tomato^29^. In addition, clear GAME11 othologs could not be identified in our *P. polyphylla* and *T. foenum–graecum* transcriptomes.

No products corresponding to mono- or polyoxygenated cholesterol were detected when *PpCYP94D108*, *PpCYP94D109*, *PpCYP72A616*, *TfCYP82J17,* or *TfCYP72A613* was expressed alone in *N. benthamiana* (Fig. 2b–d). In contrast, when each of these five genes was co-expressed with *PpCYP90G4* or *TfCYP90B50*, diosgenin was produced (Fig. 1d and Supplementary Fig. 4). The contribution of each of these CYPs to diosgenin biosynthesis is likely different across different plant tissues, evidenced by their different expression levels in various tissues of *P. polyphylla* and *T. foenum–graecum* (Supplementary Fig. 19). Together with the in vitro yeast microsome enzyme assay result (Supplementary Fig. 17), we propose that these five enzymes likely catalyze 27-monohydroxylation of **4**, which subsequently triggers stereospecific formation of the terminal heterocycle to yield diosgenin (Fig. 4). An analogy can be drawn to the dihydroxy-ketone mechanism utilized by the bacterial cyclases AveC, MeiC, and RevJ for 6,6-spiroketal production^15,16,30^. These non-redox-active enzymes stabilize an oxonium intermediate to promote stereospecific cyclization, whereas their absence results in spontaneous formation of an epimeric mixture about the spiroketal carbon^15^. Considering only a single diastereomer of diosgenin is observed in our reactions, this analogy would suggest an additional role of the second set of CYPs in promoting and stabilizing a C22 oxonium formation after canonical oxidative CYP-mediated turnover of **4** (Supplementary Fig. 20a). A more plausible proposal for the stereospecific spiroketalization of diosgenin that is consistent with CYP chemistry would invoke the dehydration of the newly installed 27-hydroxyl, thus retaining the C22 stereocenter of **4** (Fig. 4). The dehydration could be mediated within the CYP active site by the heme ferric ion acting as a lewis acid as hydroxylated products may momentarily remain coordinated post oxidation (Fig. 4). However, we cannot rule out alternative cyclization mechanisms invoking direct radical coupling or desaturation for diosgenin production (Supplementary Fig. 20b, c), although our data do support a previously unknown approach to enzymatic spiroketalization distinct from previously characterized systems^15^.

### The evolutionary origins of diosgenin biosynthesis

**Fig. 5:**
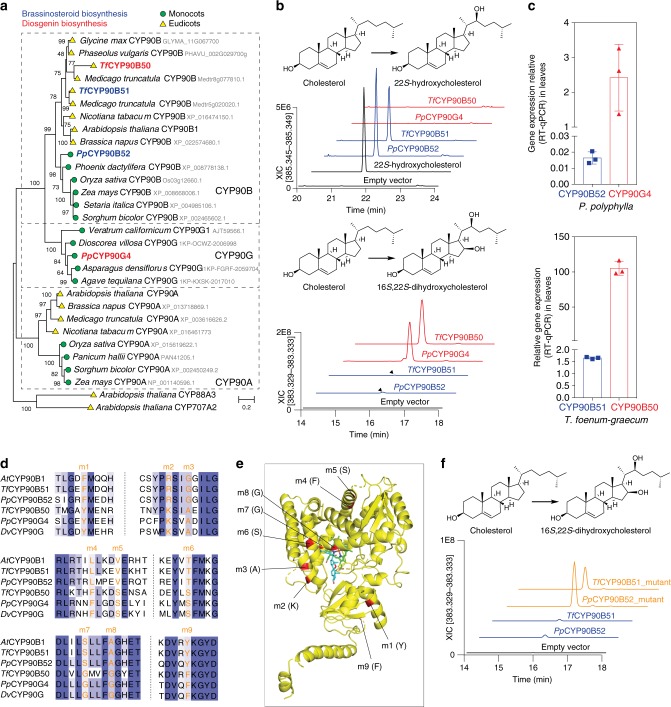
Parallel evolution of diosgenin-biosynthetic CYP90s in monocots and dicots. **a** Maximum-likelihood phylogenetic tree of CYP90-family members from various plants, rooted on *A. thaliana* CYP88A3 and CYP707A2 as the outgroup. Bootstrap values (based on 2000 replicates) are indicated at the tree nodes. The scale measures evolutionary distances in substitutions per amino acid. Protein sequences were retrieved from GenBank or 1KP^56^ with their accession numbers/loci indicated. **b** Cholesterol 22-monohydroxylase and 16,22-dihydroxylase activity of diosgenin-biosynthetic CYP90s and BR-biosynthetic CYP90Bs from *P. polyphylla* and *T. foenum–graecum*. XICs, Extracted ion chromatograms. **c** Relative transcript levels of diosgenin-biosynthetic *CYP90*s and BR-biosynthetic *CYP90*s in leaf tissues of *P. polyphylla* and *T. foenum–graecum* determined by quantitative RT-PCR. Error bars, mean ± s.d. (*n* = 3 biological replicates). Source data are provided as a Source Data file. **d** Multiple sequence alignment of canonical BR-biosynthetic CYP90s (*A. thaliana* CYP90B1 (*At*CYP90B1), *Tf*CYP90B51, and *Pp*CYP90B52) and diosgenin-biosynthetic CYP90Bs (*Tf*CYP90B50, *Pp*CYP90B52, and *Dioscorea villosa* CYP90G (*Dv*CYP90G)), highlighting nine residue positions that are differentially conserved within each sequence group. The full alignment is displayed in Supplementary Fig. 21a. **e** 3D protein model of diosgenin-biosynthetic *Tf*CYP90B50. The model was build using Phyre 2^57^. The nine residue positions that are differentially conserved between two groups of sequences are highlighted in red. *Tf*CYP90B50 was aligned in PyMOL (Schrödinger) with the crystal structure of human CYP11A1 in complex with 20,22-dihydroxycholesterol (PBD: 3NA0, (26)), of which heme and 20,22-dihydroxycholesterol are shown in green and blue, respectively (a close-up view is shown in Supplementary Fig. 21b). **f** Mutating the nine residues in *Pp*CYP90B52 and *Tf*CYP90B51 to the corresponding amino acids as in *Pp*CYP90G4 and *Tf*CYP90B50 resulted in significant increase of the cholesterol 16,22-dihydroxylase activity. The enzymatic activities were demonstrated by transient expression experiments in *N. benthamiana*, and displayed in selected reaction monitoring chromatograms (SRM) from LC–MS data

Our results demonstrate that *Pp*CYP90G4 and *Tf*CYP90B50 are key initiators of furostanol- and spirostanol-type steroid biosynthesis. To explore the evolutionary origins of these two CYP90-family enzymes, we computed a maximum-likelihood phylogenetic tree based on multiple sequence alignment of CYP90-family amino-acid sequences collected from *P. polyphylla* and *T. foenum–graecum* as well as other monocot and eudicot species (Fig. 5a). Both *Pp*CYP90G4 and *Tf*CYP90B50 share a close phylogenetic relationship with the canonical CYP90Bs, which are an orthologous group of enzymes that catalyze the rate-limiting sterol-C22 hydroxylation reaction in brassinosteroid (BR) biosynthesis^31^. BRs are essential phytohormones in land plants that regulate various aspects of plant growth and development^32^. Both *P. polyphylla* and *T. foenum–graecum* appear to contain canonical CYP90Bs, which are annotated as *Pp*CYP90B52 and *Tf*CYP90B51, respectively (Fig. 5a). Our phylogenetic analysis indicates that the paralogous diosgenin-biosynthetic *Pp*CYP90G4 and *Tf*CYP90B50 resulted from two independent gene duplication events. Whereas *Pp*CYP90G4 together with numerous CYP90s from several known diosgenin-producing monocot species forms a clade representing a relatively ancient gene duplication event that predated the monocot-dicot split, *Tf*CYP90B50 was descended from a recent gene duplication event that only occurred in legumes (Fig. 5a).

The canonical CYP90B1 from Arabidopsis catalyzes 22-monohydroxylation of C_27_, C_28_, and C_29_ sterols, including cholesterol, campesterol, and sitosterol^33^. We confirmed that *Pp*CYP90B52 and *Tf*CYP90B51 are indeed canonical BR-biosynthetic enzymes capable of converting cholesterol to 22-hydroxycholesterol when expressed in *N. benthamiana* (Fig. 5b). Interestingly, this presumably ancestral sterol 22-monohydroxylase activity is entirely suppressed in the derived diosgenin-biosynthetic paralogs *Pp*CYP90G4 and *Tf*CYP90B50 (Fig. 5b). As BR biosynthesis is tightly controlled in plants^34^, the complete suppression of the sterol 22-monohydroxylase activity in *Pp*CYP90G4 and *Tf*CYP90B50 was likely driven by strong negative selection, as any residual sterol 22-monohydroxylase activity retained in these derived diosgenin-biosynthetic CYP90s would likely disturb BR homeostasis and cause growth defects^34^. Indeed, the relative gene expression levels of *PpCYP90G4* and *TfCYP90B50* are orders of magnitude higher than those of *PpCYP90B52* and *TfCYP90B51* in their native host species (Fig. 5c), and BR homeostasis is seemingly not disturbed.

Interestingly, heterologous expression of *Pp*CYP90B52 and *Tf*CYP90B51 in *N. benthamiana* also yielded trace amounts of **1** (Fig. 5b), implicating that the additional sterol 16-hydroxylation activity necessary for diosgenin biosynthesis was probably present as a promiscuous activity in the ancestral BR-biosynthetic CYP90Bs from which the diosgenin-biosynthetic CYP90s were derived. Analyzing the sequence alignment of canonical BR-biosynthetic CYP90s and the derived diosgenin-biosynthetic CYP90s identified nine residue positions that are differentially conserved between two groups of sequences (Fig. 5d and Supplementary Fig. 21a). When mapped to a modeled structure of *Tf*CYP90B50, four of the nine residues (m2, 3, 7, and 8) are located in close proximity to the catalytic center, whereas the other five (m1, 4, 5, 6, and 9) are spread throughout the CYP structure (Fig. 5e and Supplementary Fig. 21b). To test the role of these differentially conserved residues in functional evolution within the CYP90 family, we mutated the nine residues in *Pp*CYP90B52 and *Tf*CYP90B51 to the corresponding amino acids as in *Pp*CYP90G4 and *Tf*CYP90B50. The resultant mutants, namely *Pp*CYP90B52_mutant and *Tf*CYP90B51_mutant, exhibit significantly increased cholesterol 16,22-dihydroxylase activity as their main activity when expressed in *N. benthamiana* (Fig. 5f). *Pp*CYP90B52_mutant and *Tf*CYP90B51_mutant also produced **2**–**5** in *N. benthamiana*, albeit at lower levels compared with *Pp*CYP90G4 and *Tf*CYP90B50 (Supplementary Fig. 22). It is likely that residues m7 and m8, which are located close to the predicted oxygen binding and activation site, influence the capability of the CYP to catalyze multiple oxidation steps on the same substrate. The relative abundance of **3** and **5** were also drastically reduced compared to the product profiles of *Pp*CYP90G4 and *Tf*CYP90B50 (Supplementary Fig. 22), indicating that the relative rate of the third catalytic cycle of cholesterol oxidation may be significantly slower than the subsequent cyclization step in *Pp*CYP90B52_mutant and *Tf*CYP90B51_mutant. Unlike *Pp*CYP90G4 and *Tf*CYP90B50, *Pp*CYP90B52_mutant and *Tf*CYP90B51_mutant still retain significant residual level of cholesterol 22-monohydroxylase activity (Supplementary Fig. 23), suggesting that additional mutations must have occurred independently in *Pp*CYP90G4 and *Tf*CYP90B50 to suppress the ancestral sterol 22-monohydroxylase activity. Taken together, these results illustrate that the catalytic plasticity embedded within the canonical BR-biosynthetic CYP90Bs provides the basis for independent acquisitions of novel catalytic activities towards specialized furostanol- and spirostanol-type steroid biosynthesis in distantly related plants. On the other hand, although *Pp*CYP90G4 and *Tf*CYP90B50 were descended from independent gene duplication events, they carry identical substitutions at the nine positions seemingly critical for the neofunctionalization of the cholesterol 16,22-polyhydroxylase activity from the ancestral sterol 22-monohydroxylase activity. This may suggest that the evolutionary trajectories to arrive at an optimal diosgenin-biosynthetic cholesterol 16,22-polyhydroxylase from a BR-biosynthetic CYP90B progenitor could be highly constrained, which echoes the similar observation arose from a classical experiment that explored all possible mutational trajectories underpinning the evolution of an antibiotic-resistant allele of β-lactamase^35^.

## Discussion

**Fig. 6:**
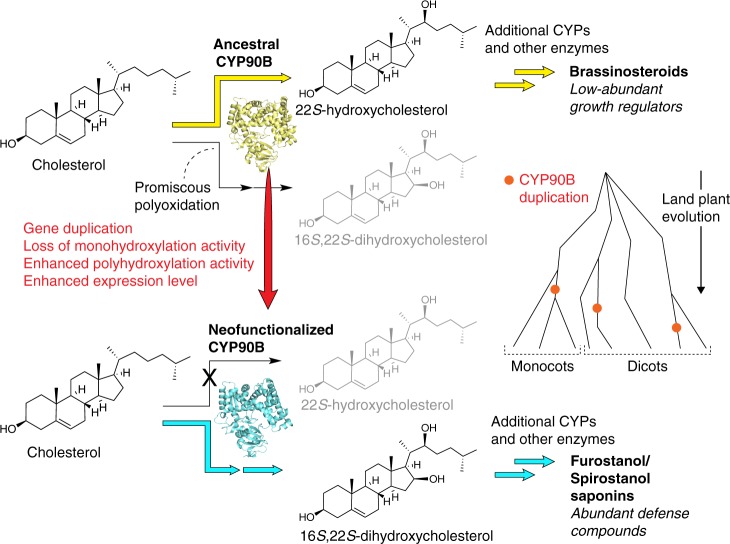
Evolutionary model for occurrences of furostanol and spirostanol biosynthesis in plants. Through independent gene duplication events followed by neofunctionalization, the latent sterol 16,22-polyhydroxylase activity contained within the ancestral brassinosteroid (BR)-biosynthetic CYP90Bs was recruited to yield 16,22-polyoxygenated cholesterols, which were further derivatized by additionally recruited enzymes to establish the evolutionarily new furostanol- and spirostanol-type steroid biosynthetic systems in certain plants. Despite their evolutionary origin in phytohormone metabolism, these CYP90s exhibit a total suppression of the ancestral sterol 22-monohydroxylase activity likely owing to negative selection, which allow them to fully excise defense function without disturbing BR homeostasis

Specialized steroidal metabolism is widespread in both animals and plants. Many steroidal metabolic traits are taxonomically restricted to only a particular lineage of organisms as the result of unique adaptation to specific environmental niches, whereas some others distribute sporadically across several lineages that are phylogenetically distant from each other, presenting potential cases of independent metabolic evolution propelled by common selective pressures^36^. The occurrences of diosgenin in distantly related monocot and dicot families separated by 150 million years of evolution illustrate the latter scenario with the underpinning metabolic pathways assembled through the mechanism of parallel evolution^36^. The promiscuous sterol 16,22-dihydroxylase activity associated with the ancestral BR-biosynthetic CYP90Bs likely sowed the seeds for the repeated emergence of CYP90 paralogs that subsequently tamed this latent activity through molecular evolution to produce new defense compounds (Fig. 6). Other cases of neofunctionalized CYP90s have been reported previously. For example, CYP90G1 from *Veratrum californicum* was shown to catalyze the conversion of the C22-hydroxyl moiety to a ketone in cyclopamine biosynthesis^37^. However, the enzymatic activity of *Pp*CYP90G4 and *Tf*CYP90B50, i.e., cholesterol 16,22-polyhydroxylation followed by cyclization, has never been described earlier.

End-of-tail hydroxylation by CYPs was previously reported in plants, e.g., in *Arabidopsis thaliana* (CYP734A1)^38^ and *V. californicum* (CYP94N1)^37^. The recruitment of a second CYP from several distinct CYP families to complete diosgenin biosynthesis in both *P. polyphylla* and *T. foenum–graecum* likely reflects the relative ease for the furostanol end-of-tail hydroxylation and cyclization chemistry to come about once the key intermediate (i.e., compound **4**) became available. The metabolic innovation of diosgenin as a new defense compound in certain plants with ancestry rooted in BR biosynthesis also highlights the intricate evolutionary process that conciliates between plant growth and defense (Fig. 6). Negative selection apparently has occurred in at least two independent cases to suppress the ancestral sterol 22-monohydroxylase activity in the derived diosgenin-biosynthetic CYP90s, allowing them to be insulated from BR metabolism and express at high level to produce high amounts of defense compounds.

De novo elucidation of specialized metabolic pathway in nonmodel plants remains challenging owing to a general lack of biosynthetic gene clusters as in bacteria and fungi^17^. We illustrate that pooled screens based on reasonable biosynthetic proposals are a powerful and broadly applicable approach to counter this challenge. Similar pooled transfection experiment was successfully employed by Takahashi and Yamanaka^39^ to identify essential factors required for inducing pluripotent stem cells. This approach is now increasingly facilitated by the rapid advances in plant genomics and synthetic biology tools. The application of the crystalline sponge technique^26^ in this study also played a critical role to help resolve the structures of key biosynthetic intermediates, which are often limited in quantity and difficult to resolve using more conventional methods. The complete reconstitution of diosgenin biosynthesis in *N. benthamiana* and yeast demonstrates the feasibility to produce diosgenin in alternative and sustainable hosts through metabolic engineering. At present, it is hard to predict whether heterologous production of diosgenin will be economically competitive with the well-established extraction process from yam species. However, incorporation of additional steroid-modifying enzymes into these synthetic systems can further expand the access to a wide array of diosgenin-derived analogs useful for downstream pharmaceutical development. Equipping such capabilities is particularly timely as we face the threat of global warming and rapid loss of suitable habitats for cultivating many economically important medicinal plants around the world.

## Methods

### Chemicals

Unless otherwise specified, chemicals used in this study were purchased from Sigma-Aldrich.

### Plant materials

Fenugreek (*Trigonella foenum–graecum*) and *Nicotiana benthamiana* were grown in a greenhouse on soil under a 16-h-light/8-h-dark photoperiod with fluorescent light of 80–120 μmol photons m^−2^ s^−1^ at 22 °C and 60% relative humidity. *Paris polyphylla var. yunnanensis* plants were grown in a local research station affiliated to the State Key Laboratory for Conservation and Utilization of Bio-Resources in Yunnan.

### Transcriptome analysis

Total RNA was extracted from different tissues of *P. polyphylla* (root, stem, leaf, and fruit) using the QIAGEN RNeasy Plant Mini Kit and sent to Novogene (https://en.novogene.com), where mRNA library preparation and RNA-seq analysis were performed according to the service provider’s standard protocol. Total RNA was extracted from different tissues of *T. foenum–graecum* (stem, leaf, flower and developing seed pods) using the QIAGEN RNeasy Plant Mini Kit and subjected to quality assessment by Agilent Bioanalyzer. Subsequent mRNA library preparation and RNA-seq analysis were performed by the Genome Technology Core at the Whitehead Institute. The library was prepared using the TruSeq Stranded Total RNA with Ribo Zero Library Preparation Kit (Illumina) and sequencing was performed on a Hiseq 2000 Illumina sequencer in HISEQRAPID mode. Raw sequences were assembled using Trinity in strand-specific mode^40^. Gene expression measured by transcripts per million was calculated using RSEM^41^. Candidate coding regions within transcript sequences were identified using Transdecoder^42^ and annotation of transcripts/predicted protein sequences was performed using in-house scripts. To extract full-length CYP candidates from Trinity-assembled transcriptomes, we used an in-house Python script that searches for transcripts containing the Pfam PF00067, having a length between 400 and 600 amino acids and having a STOP codon. CYPs selected for the screen presented in Fig. 1 were named based on sequence similarity as described^43^ and are compiled in Supplementary Dataset 2.

### Molecular biology

Genes of interest were either amplified by PCR from cDNA using primers ordered from Integrated DNA Technologies or de novo synthesized by Twist Bioscience or the Beijing Genomics Institute. All primers used in this study are listed in Supplementary Dataset 3. Q5 High-Fidelity DNA (New England BioLabs) polymerase was used for gene amplification. Genes of interest amplified by PCR or de novo synthesized were cloned into XhoI/AgeI-linearized pEAQ-HT vector^20^ by Gibson assembly following manufacturer’s instructions. We employed Golden Gate assembly to construct yeast expression vectors using the Yeast Toolkit developed by the Dueber lab as follows^44^. Bsm*BI*, Bsa*I,* and Not*I* restriction sites were removed from the genes of interest during gene synthesis or as previously described^44^. Genes were first introduced into the entry vector pYTK1 via *BsmBI* assembly. Correct clones were verified by Sanger sequencing. Subsequently, cassette plasmids were assembled via *BsaI* assembly. Finally, multigene plasmids were assembled into final yeast vectors (integration plasmid or 2-micron plasmid) in *BsmBI* assemblies. Correct clones were verified by diagnostic restriction digest. All plasmids constructed in this study are listed in Supplementary Dataset 1. Yeast transformation was performed as previously described^45^. Yeast strains used in this study are listed in Supplementary Table 3.

### Phylogenetic analyses

Phylogenetic trees were built using MEGA6^46^ as indicated in figure legends using either Neighbor-Joining or Maximum-likelihood methods. Bootstrap values (based on 1000 or 2000 replicates) are indicated at the tree nodes.

### qRT-PCR analysis

Total RNA was isolated as described above and cDNA was synthesized using the SuperScript III Reverse Transcriptase with Oligo dT primers (Thermo Fisher Scientific). qRT-PCR analysis were performed as previously described^47^ on a QuantStudio 6 Flex system (Thermo Fisher Scientific) using SYBR Green Master Mix (Thermo Fisher Scientific). Primers used are listed in Supplementary Dataset 3. Gene expression values were calculated using Ct values and normalized using the following housekeeping genes: *P. polyphylla*, closest homolog to *Hordeum vulgare* alpha tubulin U40042.1^48^; *T. foenum–graecum*, closest homolog to *Medicago truncatula* Medtr3g091400.1^49^.

### Transient expression of *CYP*s in *N. benthamiana*

Transient expression of CYPs in *N. benthamiana* was performed as follows. pEAQ-HT vectors^20^ carrying candidate *CYP* genes were transformed into *A. tumefaciens* LBA4404-competent cells by electroporation (2.5 kV, cuvettes with 0.1 cm gap). Transformants were selected on YM medium (for 1 L: 0.4 g yeast extract, 10 g of mannitol, 0.1 g of sodium chloride, 0.2 g of magnesium sulfate (heptahydrate), 0.5 g of potassium phosphate (dibasic, trihydrate), 15 g of agar, pH 7 with KOH) supplemented with 100 µg/mL rifampicin, 50 µg/mL kanamycin, and 100 µg/mL streptomycin. Agrobacterium colonies were inoculated in liquid YM medium containing 100 µg/mL rifampicin, 50 µg/mL kanamycin, and 100 µg/mL streptomycin, and incubated at 30 °C with shaking at 225 rpm for 24–36 h. These cultures were then diluted in larger volumes (1:10) and further grown for 24 h. Cells were collected by centrifugation at 3000 × *g* for 30 min and cell pellets were washed once with 20 mL of MMA buffer (10 mm MES (pH 5.6 with KOH), 10 mm magnesium chloride, 100 µm acetosyringone). The washed cells were resuspended in MMA buffer to reach an optical density (OD) of 0.8. For pooled *Agrobacterium* infiltrations, equal volume of each strain suspension were mixed together.

Prior to infiltration, *N. benthamiana* plants (6 weeks old) were watered and placed in the shade for ~ 2 h. After infiltration, plants were kept in the shade and transferred to normal growth condition the following day. For each experiment, an Agrobacterium strain carrying the empty pEAQ-HT vector was included as a control. In total, 100 mg leaf samples were collected 5 d after infiltration in 2 mL plastic tubes containing ~ 10 methanol-washed 2 mm zirconia beads (Research Products International (9837)), immediately snap-frozen in liquid nitrogen and stored at − 80 °C until analysis.

### Cholesterol-derived metabolite extraction

Different extraction procedures were employed depending on the experiment and sample types. For the screening experiment shown in Fig. 1, diosgenin was extracted from *N. benthamiana* samples using isopropanol as follows. Frozen leaf samples were ground using TissueLyser II (Qiagen) (30 Hz for 3 min). 1 mL of isopropanol was then added to the pulverized sample, and the resulting slurry was homogenized using TissueLyser II (Qiagen) (30 Hz for 3 min). The extracts were incubated at 60 °C with shaking at 1400 rpm on a Thermomixer (Eppendorf) for 20 min, and sonicated for 20 min. Debris and insoluble materials were removed using two rounds of centrifugation at 20,000 g for 3 min. Relative concentration of diosgenin in the supernatant was measured by LC-(HR)MS as described below.

Except for the experiment presented in Fig. 1 (see above), samples from *N. benthamiana*, *P. polyphylla,* and *T. foenum–graecum* were extracted as follows. Frozen samples were thawed and homogenized in 300 µL of 100% methanol (supplemented with 0.1 mg/mL butylated hydroxytoluene (BHT)) using TissueLyser II (Qiagen) (30 Hz for 3 min). Homogenized samples were then transferred to 2 mL glass tubes (Analytical Sales and Services (tubes, 96F620, caps 96FSC2-12)). Remaining homogenized material was collected from the plastic tube using 100 µL of 100% methanol and transferred to the same glass tube. In total, 200 µL of 100% chloroform (supplemented with 0.1 mg/mL BHT) was added to the glass tube, and the sample suspension was sonicated in a sonication bath for 5 min. Samples were centrifuged at 1,000 g for 3 min and the resultant supernatant was transferred to a new glass tube. Pellets were re-extracted using 600 µL of methanol:chloroform (2:1, v/v; supplemented with 0.1 mg/mL BHT) and sonication for 5 min. Samples were centrifuged at 1000 g for 3 min and the resultant supernatants were combined with the supernatant from the first extraction. Combined supernatants were then dried using nitrogen gas. Dried samples were then saponified in 300 µL methanol:10% KOH (8:2, v/v; final KOH concentration 2%; supplemented with 0.1 mg/mL BHT) for 1 h at room 22 °C with shaking at 150 rpm. 600 µL of water was then added to the saponified samples, and the unsaponifiable lipids were extracted three times with 300 µL of hexanes. The hexane fractions were pooled into a new glass tube, wash three times with 300 µL of water and then dried using nitrogen gas. Triterpenoids were resuspended in 100 µL of 100% methanol. Glass tubes were sonicated 5 min in a sonication bath, and insoluble compounds were removed by centrifugation (10,000 g in plastic tubes) before analysis by LC-(HR)MS as described below.

To extract sterols from yeast, 1 mL sample of yeast culture was centrifuged at 10,000 g for 2 min to collect the yeast cells. Cells were then resuspended in 300 µL methanol:10% KOH (8:2, v/v; final KOH concentration, 2%; supplemented with 0.1 mg/mL BHT) and then lysed using TissueLyser II (Qiagen) with 2 mm zirconia beads (~ 10 beads per tubes; Research Products International (9837)) (30 Hz for 3 min). Samples were then incubated for 1 h at room 22 °C with shaking (150 rpm) to allow saponification of the samples. In total, 600 µL of water was then added to the saponified samples, and unsaponifiable lipids were extracted three times with 300 µL of hexane. Hexane fractions were pooled into a new glass tube, wash three times with 300 µL of water and then dried using nitrogen gas. Triterpenoids were resuspended in 100 µL of 100% methanol. Glass tubes were sonicated 5 min in a sonication bath and insoluble compounds were removed by centrifugation (10,000 g in plastic tubes) before analysis by LC-(HR)MS.

### Microsome assays

Yeast microsomes were prepared as previously described with a few modifications^50^. In brief, cells were grown in YPD (YP + Dextrose) to stationary phase and diluted into YPG (YP + 2% Galactose) in the presence of 0.1 mm CuSO_4_ for overnight induction at 30 °C. Approximately, 120 ODs of cells were collected and washed once with 15 ml of lysis buffer containing 15% glycerol, 50 mm K_3_PO_4_ (pH 7.4), 5 mm EDTA, 1 mm PMSF, and 1 mm DTT. Cells were disrupted by bead-beating. Unbroken cells and debris were removed after centrifugation at 3000 g at 4 °C for 5 min. The microsome fraction was prepared by ultracentrifugation at 100,000 g at 4 °C for 50 min (Beckman Coulter MLA-130). The cytosolic fraction was discarded and the membrane fraction was resuspended in lysis buffer and was snap-freezed by liquid N_2_. Microsomes prepared from different strains were mixed as described in Supplementary Fig. 17 in the presence of 50 mm NADPH and 50 mm MgCl_2_ on the ice and were subsequently incubated at 30 °C. The reaction was terminated as indicated time by mixing with 5 × volume of ethanol. The cholesterol-derived metabolites were extracted with hexane as described above. The assays were analyzed by LC–MS as described below.

### Metabolomic profiling

LC–MS was performed on a TSQ Quantum Access MAX triple-quadrupole mass spectrometer (Thermo Scientific) coupled to an Ultimate 3000 Rapid Separation LC system (Thermo Scientific). A Q-Exactive mass spectrometer (Thermo Scientific) coupled to Ultimate 3000 Rapid Separation LC system (Thermo Scientific) was used for liquid chromatography-high-resolution mass spectrometry (LC-HRMS) analysis.

For LC–MS and LC–HRMS analyses, the reverse-phase chromatography system consisted of a 150 mm C18 column (Kinetex 2.6 µm silica core shell C18 100 Å pore, Phenomenex), which was developed using Optima™ LC/MS solvents (Fisher Chemical) with a gradient (flow rate of 0.5 mL min^−1^) of solvent B (methanol with 5 mm ammonium acetate) and in solvent A (water with 5 mm ammonium acetate) as follows (all (v/v)): 65% for 2 min, 65–99% in 25 min, 99% for 10 min, 99–65% in 0.5 min and 65% for 1.5 min.

For LC–MS analysis, the TSQ Quantum Access MAX triple-quadrupole mass spectrometer (Thermo Scientific) was configured to perform selected reaction monitoring scans with the following ion source parameters: spray voltage (+) at 3000 V, capillary temperature at 275 °C, sheath gas at 40 arb units, aux gas at 15 arb units, spare gas at 1 arb unit, max spray current at 100 (μA), probe heater temp at 350 °C, ion source: HESI-II. Diosgenin: precursor ion selection at 415.3 m/z on positive ion mode, fragmentation at 20 V, and product ion selection at 271.2 m/z; 16*S*,22*S*-dihydroxycholesterol (compound **1**): precursor ion selection at 383.3 m/z on positive ion mode, fragmentation at 20 V, and product ion selection at 365.3 m/z; Compounds **2** and **4**: precursor ion selection at 399.3 m/z on positive ion mode, fragmentation at 20 V, and product ion selection at 271.2 m/z. The m/z resolution of Q1 was set to 0.4 FWHM, the nitrogen collision gas pressure of Q2 was set to 1.5 mTorr, and the Q3 scan width was set to 0.500 m/z.

For LC-HRMS analysis, the ESI-Q-Exactive Orbitrap mass spectrometer (Thermo Scientific) was operated in full-scan mode (Scan range: 200–700 m/z; Resolution, 70,000; AGC target, 3e6; Maximum IT, 200 ms) with the following ion source parameters: spray voltage (+) at 3000 V, capillary temperature at 275 °C, sheath gas at 40 arb units, aux gas at 15 arb units, spare gas at 1 arb unit, max spray current at 100 μA, probe heater temp at 350 °C, ion source: HESI-II. For collecting fragmentation pattern, the mass spectrometer was operated in full-scan mode (Scan range: 200 to 700 m/z; Resolution, 70,000; AGC target, 3E6; Maximum IT, 120 ms) with data-dependent MS^2^ (Resolution, 17,500; AGC target, 2E5; Maximum IT, 200 ms; Loop count, 4; Isolation windows, 2 m/z; Stepped NCE: 15, 25, 35; Minimum AGC target, 2E3; Intensity threshold, 1E4; Apex trigger, off; Charge exclusion, off; Peptide match; preferred; Exclude isotopes, on; Dynamic exclusion, 10 s). XCalibur (Thermo Fisher) and MZmine 2^51^ were used to process the raw data.

### Purification of compound 1

Cholesterol-producing yeast RH6829 strain^25^ co-expressing *Pp*CYP90G4 and an *Arabidopsis CYP reductase* (Supplementary Table 3, strain JKW-35.29) was initially grown in YP medium containing 2% raffinose, and subsequently induced in 12 L of YP medium containing 2% galactose and 100 µm CuSO_4_. Cells were collected by centrifugation once the OD reached 5. Cell pellets were freeze-dried and resuspended in 1 L of 80% ethanol containing 10% KOH and 0.5 mg/mL BHT, sonicated for 15 min, and incubated at 50 °C with constant mixing. The extracts were mixed with 2 L of H_2_O and partitioned with hexane (3 × 1 L). The hexane fractions were pooled, washed twice with 2 L of H_2_O, and dried using a rotary evaporator. The dried extracts were resuspended in a minimal amount of chloroform/hexane (1:1) and separated using a silica column packed with Silica Gel 60 (EMD Chemicals Inc.). A gradient of hexane/chloroform/methanol (from 1:1:0 to 0:1:0 to 0:0:1) was used as the mobile phase. The eluate was collected with a fraction collector and subjected to LC–MS analysis as described above. The fractions enriched with **1** were pooled, dried using a rotary evaporator, and further purified using a preparative HPLC system consisting of a Shimadzu system with a LC-20AP pump, a SPD-20A UV-VIS detector and a FRC-10A fraction collector. 1.5 mg of **1** was obtained.

### Crystalline sponge (CS) analysis of compound 1

The CS [(ZnCl_2_)_3_(tpt)_2_·*x*(*tert*-butyl methyl ether)] [tpt = 2,4,6-tri(4-pyridyl)-1,3,5-triazine] was prepared as previously described^52^. The guest (compound **1**) inclusion into the porous crystal was conducted using a screw-top microvial (BGB Analytik, cat. no. LP-11090620), a screw cap with a septum seal (La-Pha-Pack, cat. no. 09 04 1533), and a syringe needle (BD, cat. no. 305165). A single crystal of the crystalline sponge was immersed in 50 µL of acetone, and treated with 5 μL of acetone containing 5 μg of compound **1**. The solvent evaporation was conducted at 50 °C for 24 h by piercing the septum with a needle. Upon completion of guest-soaking, the crystal was visually inspected and selected using a microscope with a polarizer. The dried crystals were stored at RT for 4 d and subjected to the single-crystal X-ray diffraction measurement. Single-crystal X-ray diffraction was performed on a Bruker D8 Venture Kappa DUO four-circle diffractometer equipped with an IμS micro-focus sealed tube (Cu Kα radiation), a Bruker Photon2 CPAD and a low temperature system using cold nitrogen stream (100 K). The X-ray instrument is hosted at the Department of Chemistry X-ray Diffraction Facility, Massachusetts Institute of Technology.

The raw data were processed with the SAINT and SADABS programs (Bruker). All crystal structures were solved using SHELXT (ver. 2018/2)^53^ and refined using SHELXL (ver. 2018/3)^54^. All non-hydrogen atoms were refined anisotropically. All hydrogen atoms were grown using the proper HFIX commands and refined isotropically using the riding model. Several zinc and chlorine atoms in the framework were refined with EADP commands. Minimum numbers of restraints were applied without changing the standard deviation. Solvent molecules in the pores were found in the difference electron density map and refined using the restraints (DFIX, DANG, SIMU, and ISOR). These molecules were expected to be severely disordered as a consequence of their high thermal motion and had an averaged structure of various geometry and orientation. DFIX commands were therefore applied to the refinement of the solvent molecules.

An asymmetric unit is represented in Fig. 3a. Three guest molecules of **1** (a–c), six acetone molecules and two water oxygens were found in the asymmetric unit. All of the guest molecules were ordered at the general binding sites of the CS, and have an occupancy of 100%. The restraints used for the refinement are summarized in Supplementary Fig. 6. All of the three guest molecules of **1** were refined with applying SIMU (for the whole molecules). The geometry of B was related with applying SAME for C (for the whole molecule). The crystallographic parameters are summarized in Supplementary Table 1.

Considerably large void remained in the crystal structure, and we assumed that the void was filled with disordered solvent molecules. However, we could not make a suitable model for the refinement of disordered solvent molecules due to messy electron density peaks with amplitude of < 1.0 eÅ^–3^ on the d-Fourier map. Therefore, the least square refinement at the last stage was performed using the reflection data modified by *PLATON* SQUEEZE^55^ program.

### NMR analysis of compound 1

^1^H, ^13^C, DEPT, HSQC, HMBC NMR analyses of compound 1 were performed on a Bruker AVANCE-400 NMR spectrometer with a Spectro Spin superconducting magnet. NOESY analysis was performed on a Bruker AVANCE-600 NMR with a Magnex Scientific superconducting actively-shielded magnet. Both instruments are hosted at the Department of Chemistry Instrumentation Facility, Massachusetts Institute of Technology.

### Reporting summary

Further information on research design is available in the Nature Research Reporting Summary linked to this article.

## Supplementary information

Supplementary Information

Description of Additional Supplementary Files

Supplementary Data 1

Supplementary Data 2

Supplementary Data 3

Reporting Summary

Source Data

## Data Availability

The *P. polyphylla* and *T. foenum–graecum* transcriptomes reported in this study have been deposited in the National Center for Biotechnology Information (NCBI) Sequence Read Archive (SRA) under the accession numbers SRR9118488, SRR9118489, SRR9118490, SRR9118491, SRR9118496, SRR9118497, SRR9118498, and SRR9118499, and Transcriptome Shotgun Assembly (TSA) under the accession numbers GHNI00000000, GHNH00000000, GHNG00000000, GHNE00000000, GHND00000000, GHNB00000000, GHNA00000000, and GHMT00000000. The sequences of *P. polyphylla* and *T. foenum–graecum CYP* genes characterized in this article are deposited in NCBI GenBank under the accession numbers MK636701–MK636709. The crystal structure data are archived at the Cambridge Crystallographic Data Centre under the reference number CCDC 1899808. The raw data underlying Fig. 5c and Supplementary Fig. 19 are provided as a Source Data file. Other data that support the findings of this study are available from the corresponding author upon reasonable request.

## Code availability

Codes used to assemble the transcriptomes and extract CYP sequences are available on request.
