# Supplementary material for: Repeated evolution of cytochrome P450-mediated spiroketal steroid biosynthesis in plants
Source: Nat Commun. 2019 Jul 19;10:3206. doi: 10.1038/s41467-019-11286-7 (PMC6642093; doi:10.1038/s41467-019-11286-7)
Supplement: Supplementary file 1 — Supplementary Information [file 41467_2019_11286_MOESM1_ESM.pdf]

Supplementary Information for

Repeated evolution of cytochrome P450-mediated spiroketal steroid biosynthesis in plants

Christ et al.

†Corresponding author. E-mail: wengj@wi.mit.edu

Supplementary Figures

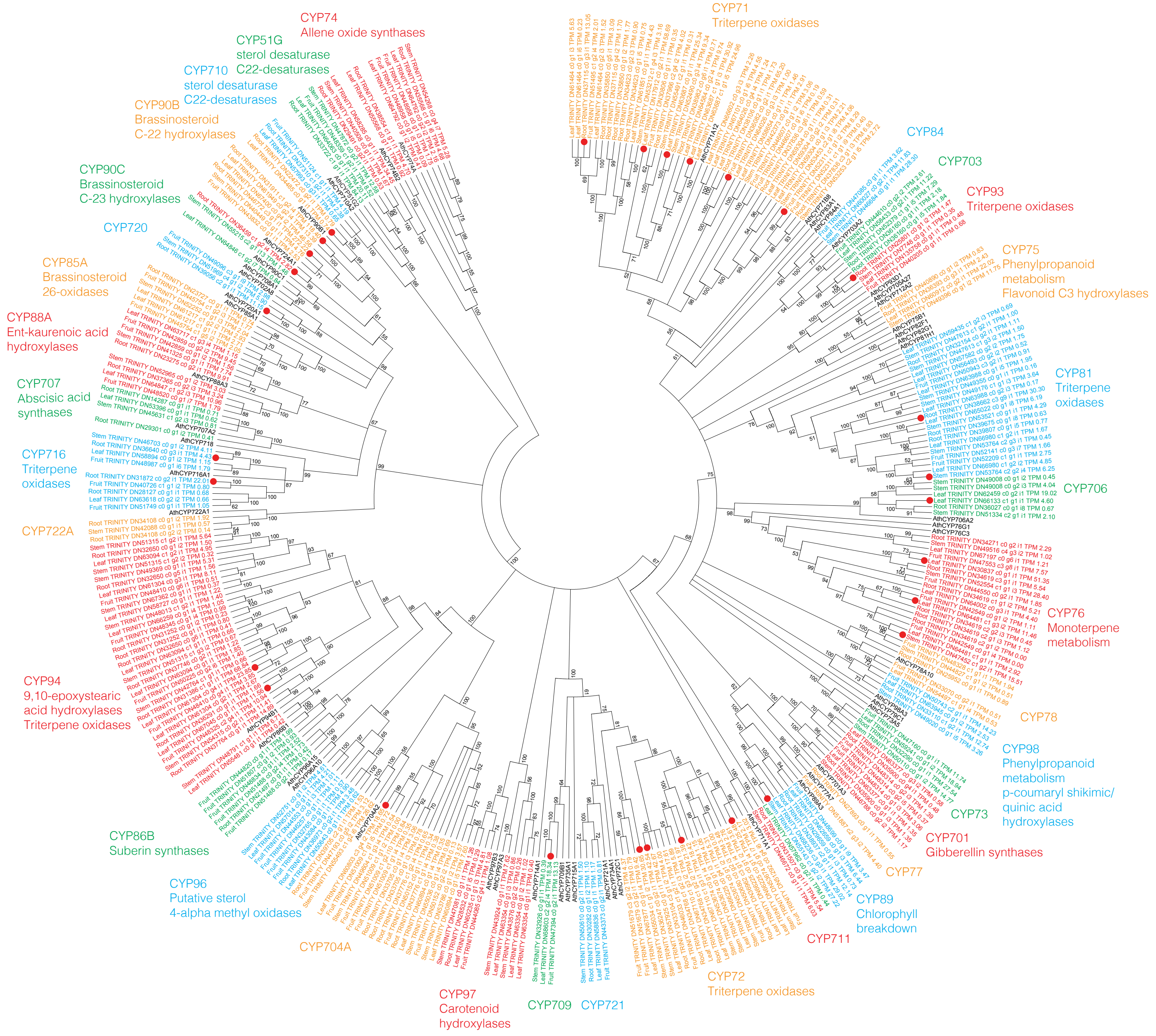

Supplementary Figure 1. Phylogenetic tree of *Paris polyphylla* CYPs. CYPs were extracted from de novo assembled transcriptomes from leaf, stem, fruit and root tissues. Selected Arabidopsis CYPs were included in the analysis to define CYP families. The 29 *Pp*CYPs selected for analysis are marked with red dots. The evolutionary history was inferred using the Neighbor-Joining method. The bootstrap consensus tree inferred from 1000 replicates is taken to represent the evolutionary history of the taxa analyzed[1]. Branches corresponding to partitions reproduced in less than 50% bootstrap replicates are collapsed. The evolutionary distances were computed using the Poisson correction method[2] and are in the units of the number of amino acid substitutions per site. The analysis involved 355 amino acid sequences. All positions with less than 95% site coverage were eliminated. That is, fewer than 5% alignment gaps, missing data, and ambiguous bases were allowed at any position. There were a total of 316 positions in the final dataset. Evolutionary analyses were conducted in MEGA6[3].

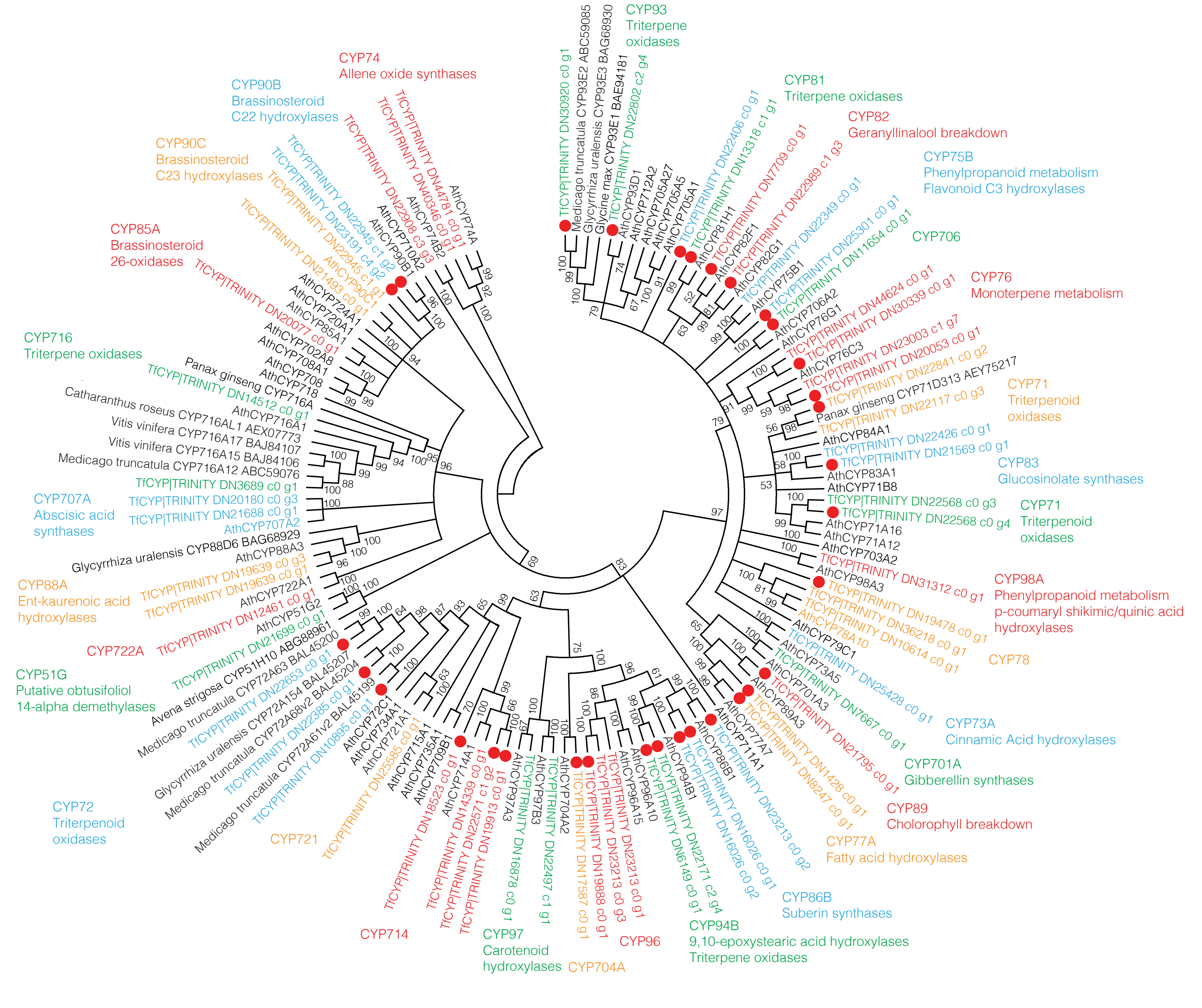

Supplementary Figure 2. Phylogenetic tree of *Trigonella foenum-graecum* CYPs. CYPs were extracted from de novo assembled transcriptomes from developing seed pods. Selected CYPs from Arabidopsis and other species were included in the analysis to define CYP families. The 33 *Tf*CYPs selected for analysis are marked with red dots. The evolutionary history was inferred using the Neighbor-Joining method. The bootstrap consensus tree inferred from 1000 replicates is taken to represent the evolutionary history of the taxa analyzed[1]. Branches corresponding to partitions reproduced in less than 50% bootstrap replicates are collapsed. The evolutionary distances were computed using the Poisson correction method[2] and are in the units of the number of amino acid substitutions per site. The analysis involved 135 amino acid sequences. All positions containing gaps and missing data were eliminated. There were a total of 280 positions in the final dataset. Evolutionary analyses were conducted in MEGA6[3].

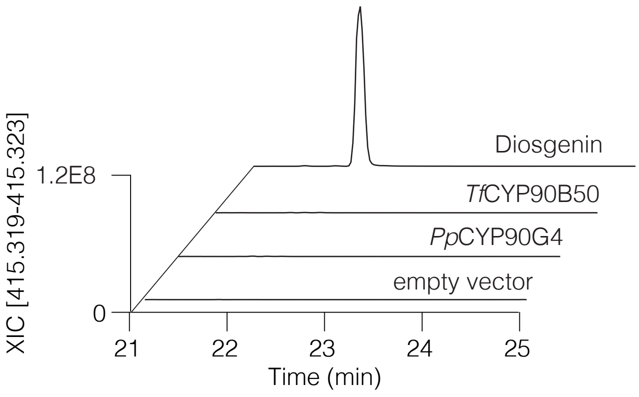

Supplementary Figure 3. Heterologous expression of *Tf*CYP90B50 and *Pp*CYP90G4 in *N. benthamiana*. Samples were analyzed by liquid chromatography-high-resolution mass spectrometry (LC-HRMS). Extracted ion chomatograms (XIC) corresponding to the mass of diosgenin ionized as $[M+H]^+$ are shown. Pure diosgenin was used as standard.

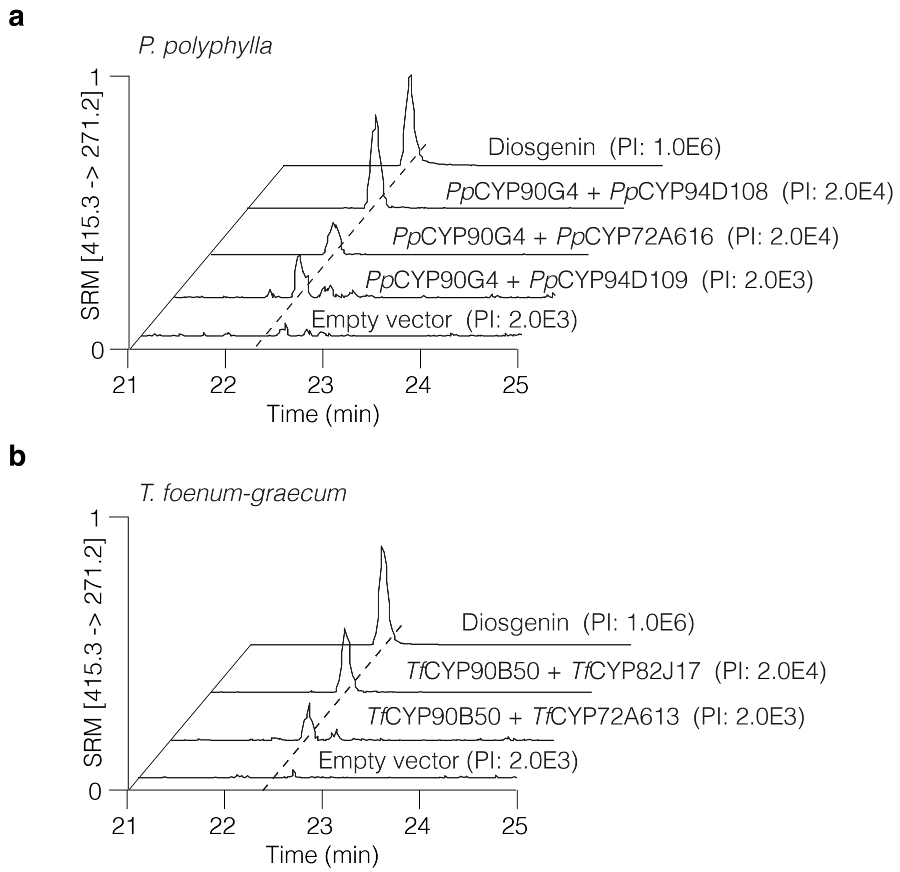

Supplementary Figure 4. Heterologous expression of identified CYP pairs (Figure 1) in *N. benthamiana*. Chromatograms derived from *P. polyphylla* CYPs are shown in (**a**). Chromatograms derived from *T. foenum-graecum* CYPs are shown in (**b**). Samples were analyzed by liquid chromatography-mass spectrometry (LC-MS). Pure diosgenin was used as standard. For clarity, peak heights are not proportional and the intensity of each peak is displayed on the right (PI, product ion intensity). SRM, selected reaction monitoring.

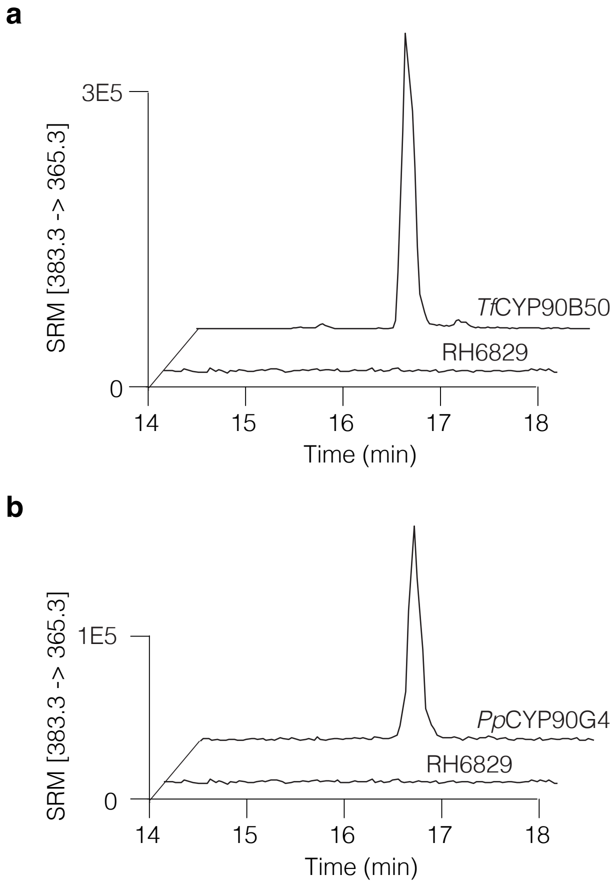

Supplementary Figure 5. Heterologous expression of CYP90Bs in yeast strain RH6829. Samples from RH6829 transformed with *Tf*CYP90B50 (**a**) and *Pp*CYP90G4 (**b**) were analyzed by liquid chromatography-mass spectrometry (LC-MS). SRM, selected reaction monitoring.

SIMU for All

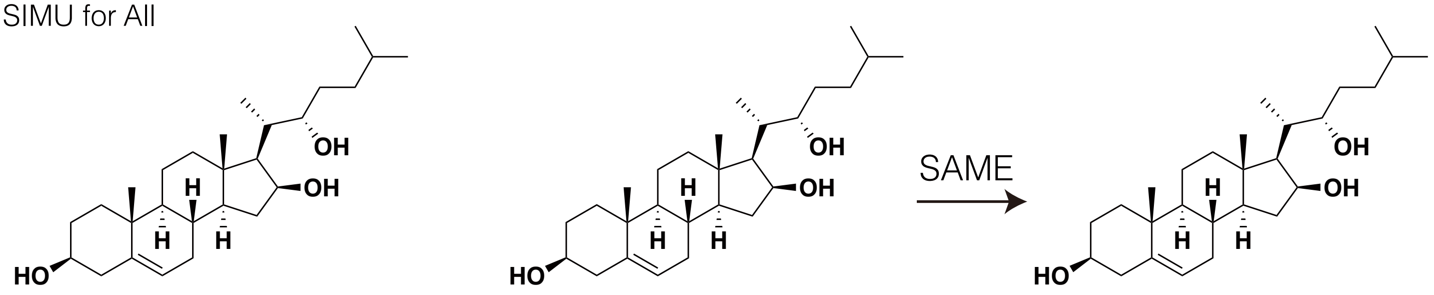

Guest ***a*** (RESI 10, Occ. 100%) Guest ***b*** (RESI 9, Occ. 100%) Guest ***c*** (RESI 11, Occ. 100%)

Supplementary Figure 6. Restraints applied in the refinement of crystalline-sponge-compound-1 complex. All of the three guest molecules of 1 were refined with applying SHELXL SIMU command (for the whole molecules). The geometry of ***b*** was related with applying SHELXL SAME command for ***c*** (for the whole molecule). The crystallographic parameters are summarized in Supplementary Table 1. See Methods section for more information.

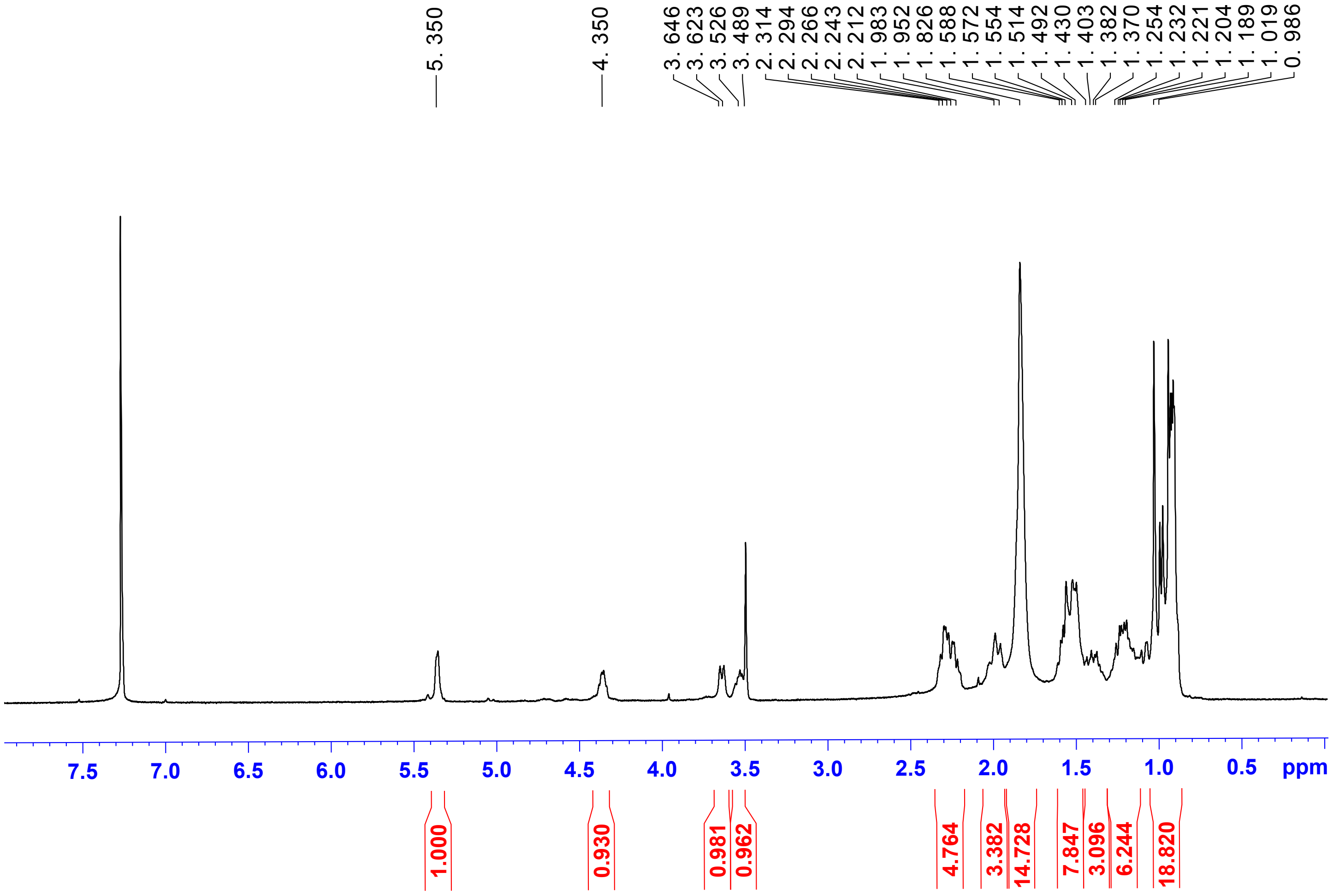

Supplementary Figure 7. ^{1}H NMR spectrum (400 MHz, $CDCl_3$) of (16S,22S)-dihydroxycholesterol.

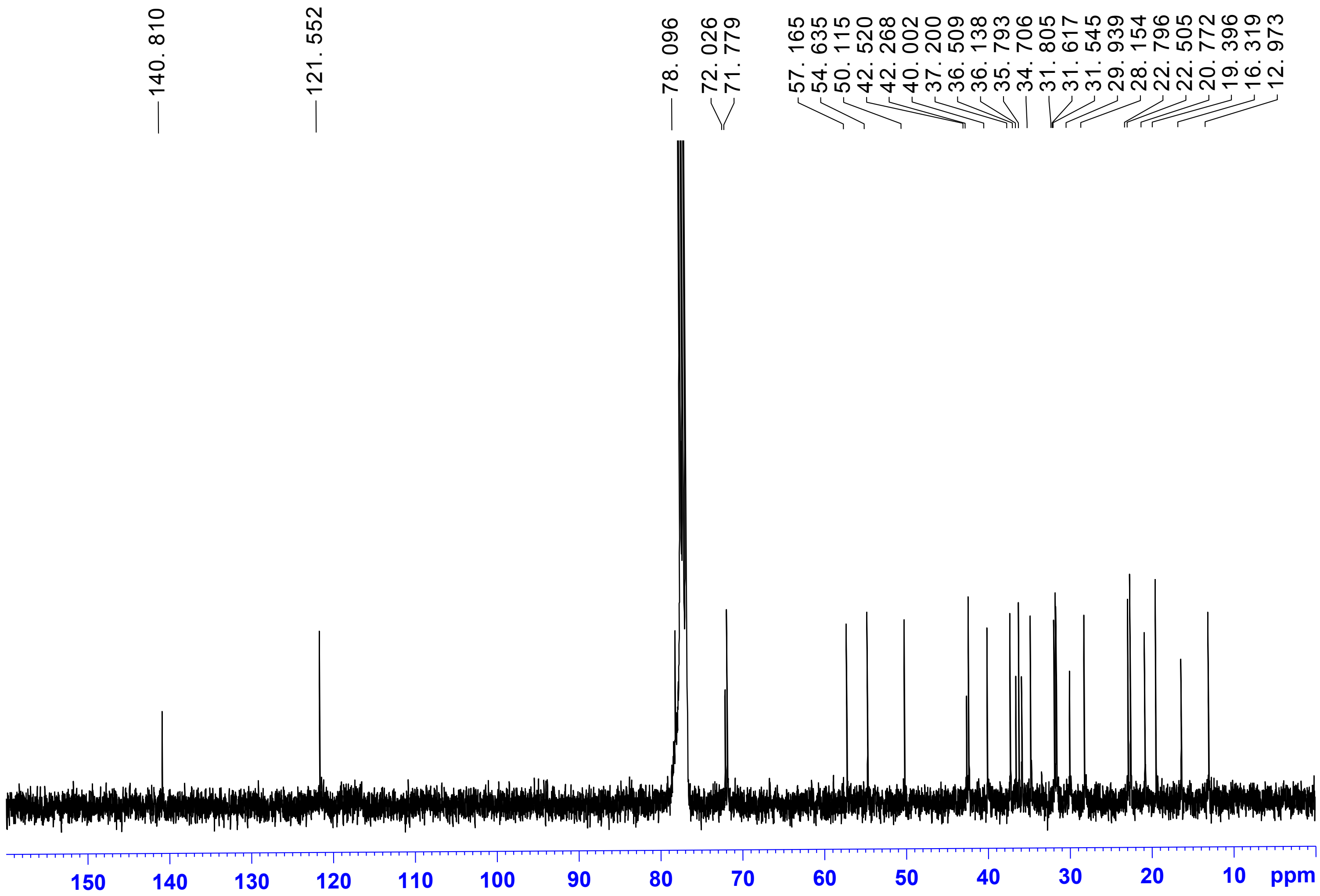

Supplementary Figure 8. ^{13}C NMR spectrum (100 MHz, $CDCl_3$) of (16S,22S)-dihydroxycholesterol.

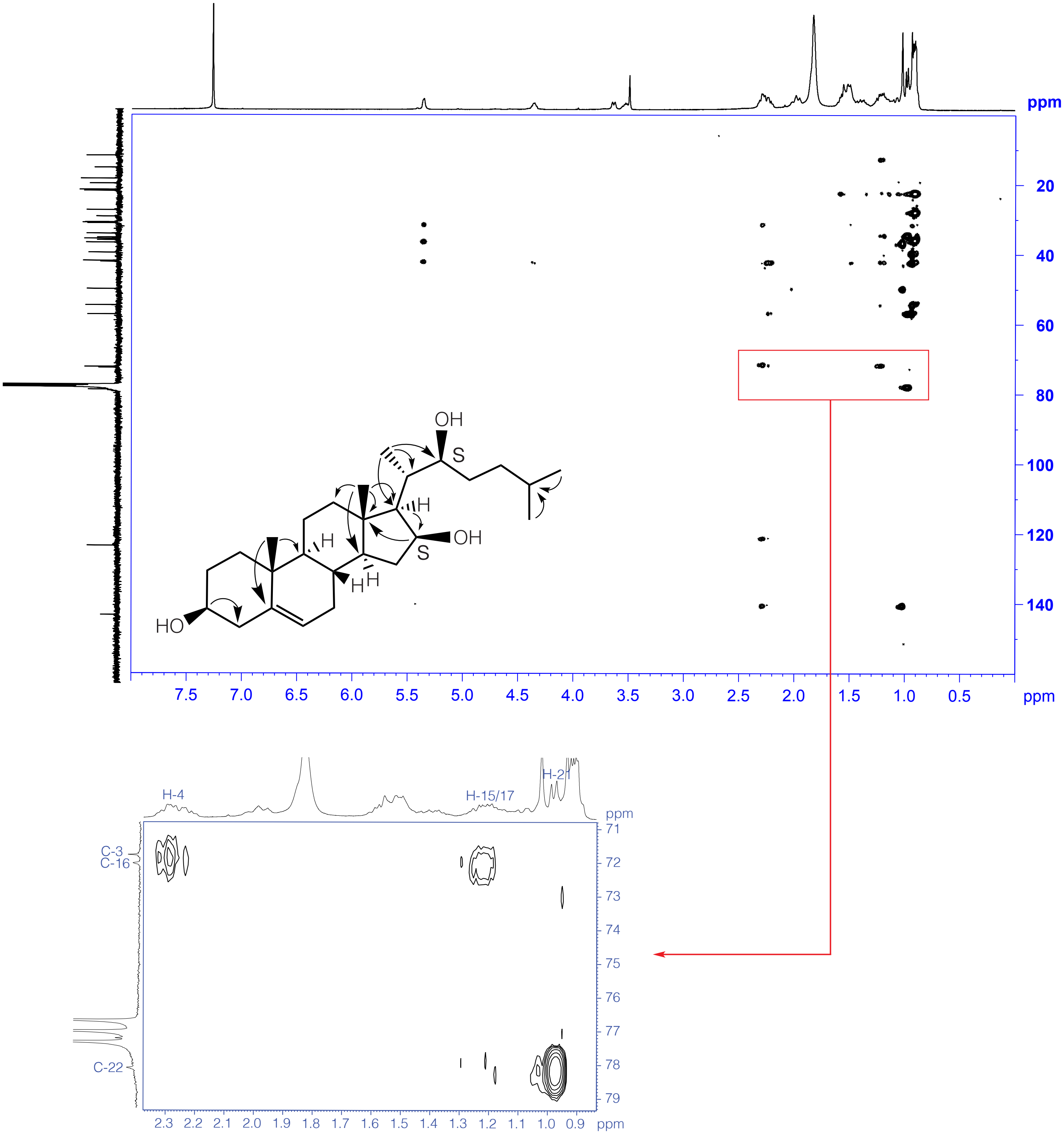

Supplementary Figure 9. HMBC spectrum of (16S,22S)-dihydroxycholesterol. A close-up view for the region depicted in red in shown.

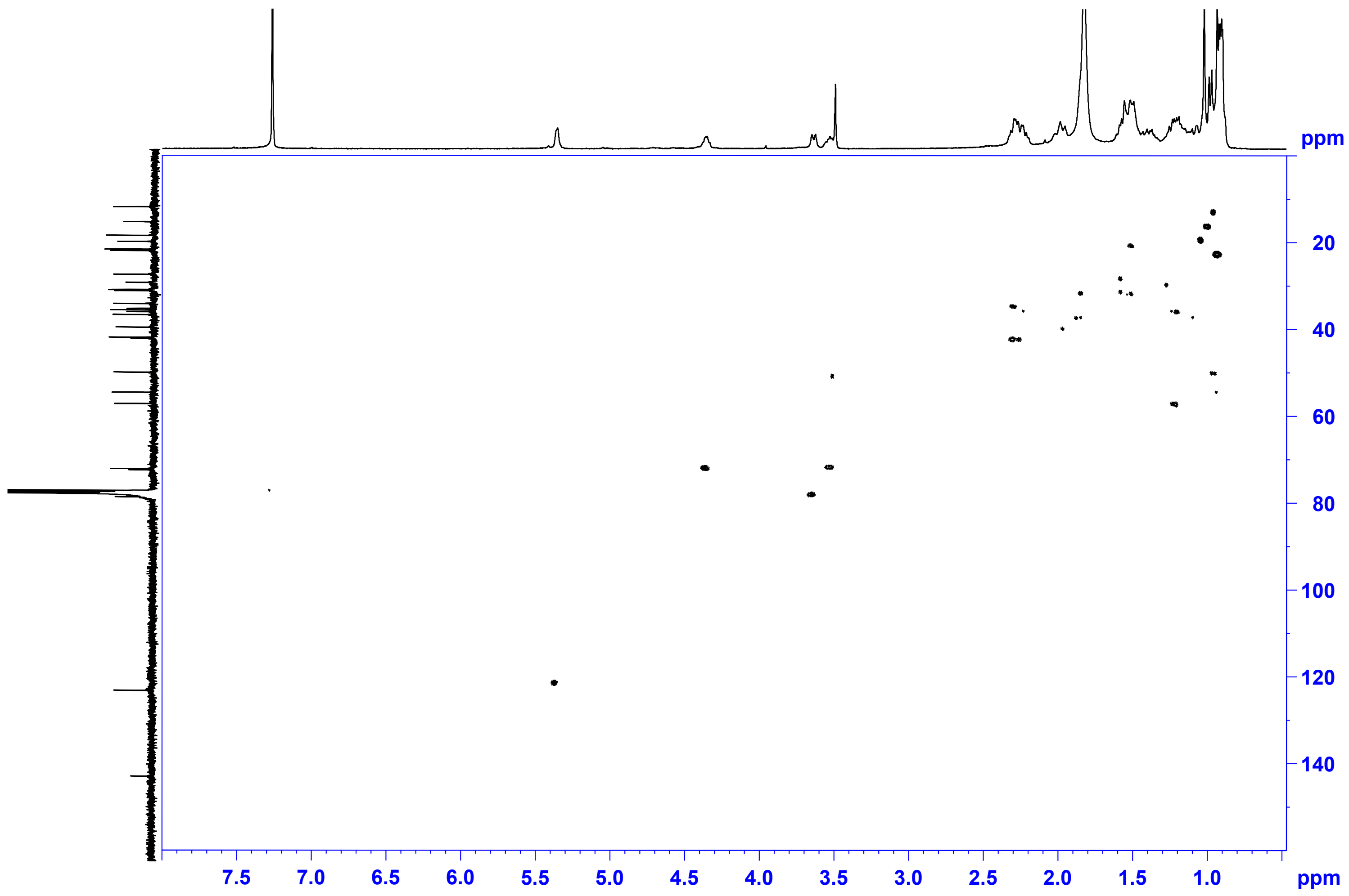

Supplementary Figure 10. HSQC spectrum of (16S,22S)-dihydroxycholesterol.

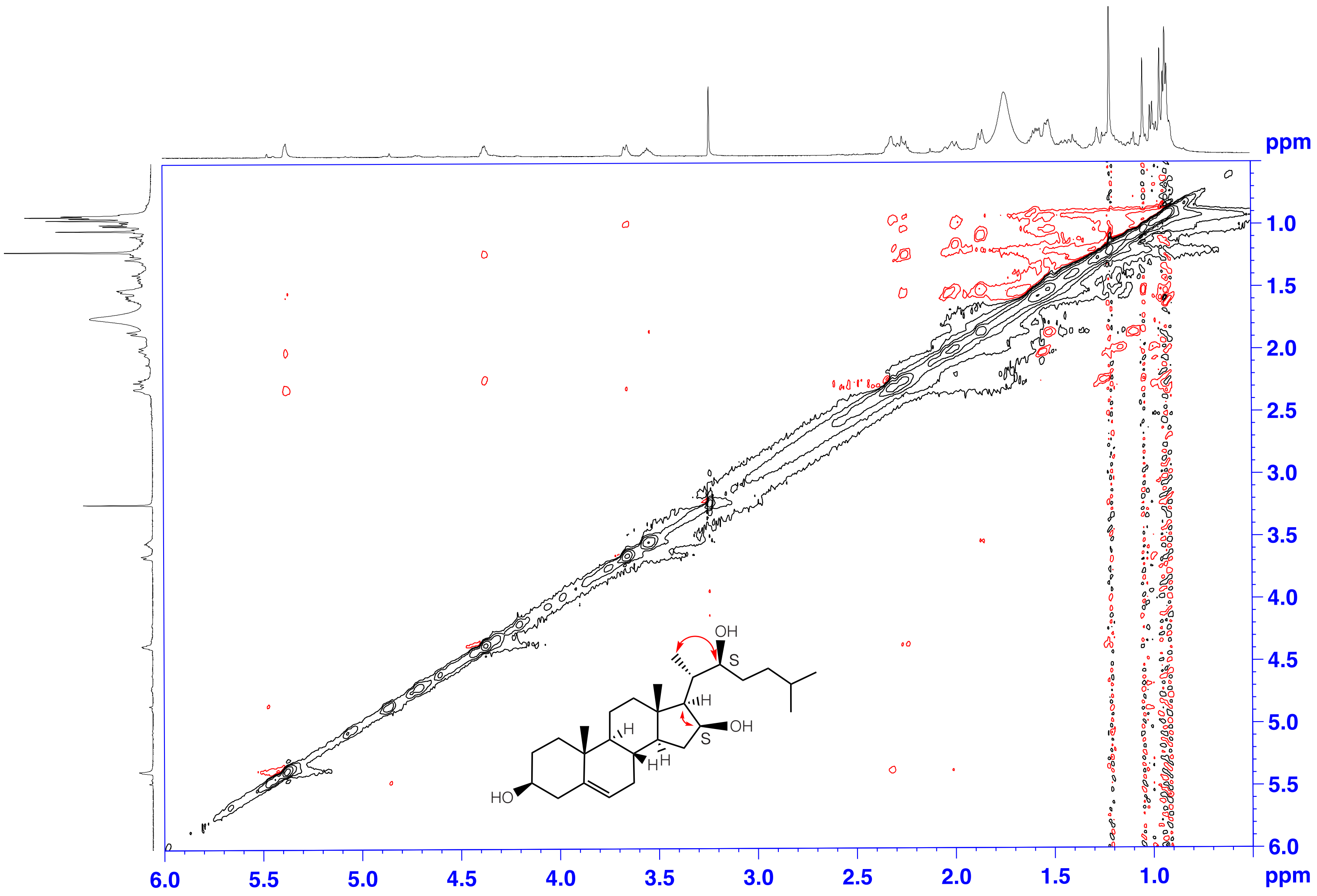

Supplementary Figure 11. NOESY spectrum of (16S,22S)-dihydroxycholesterol.

a

Compound 1, RT 15.9 min

$M=C_{27}H_{46}O_3 / P^+=C_{27}H_{47}O_3$ [M+H]$^+$

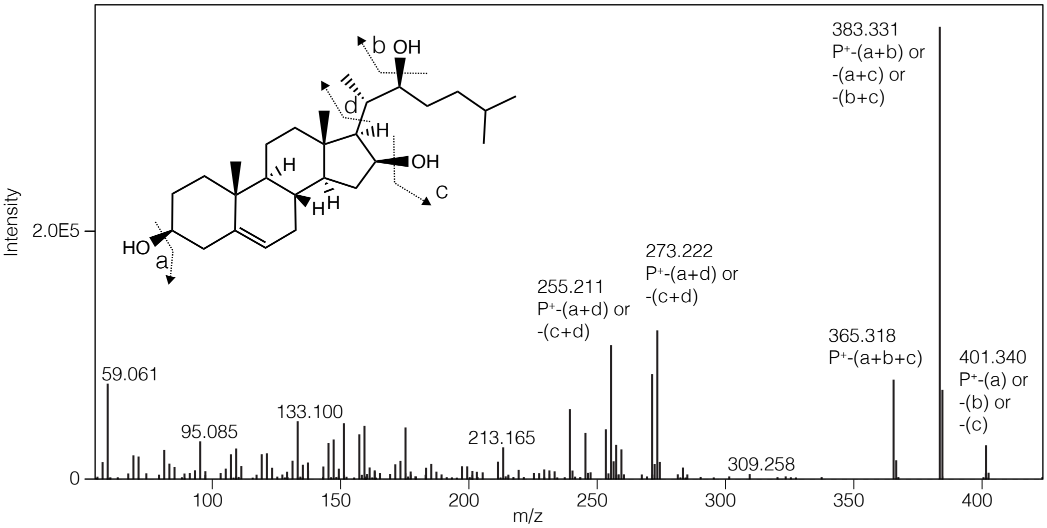

b

Compound 2, RT 24.0 min

$M=C_{27}H_{44}O_3 / P^+=C_{27}H_{45}O_3$ [M+H]$^+$

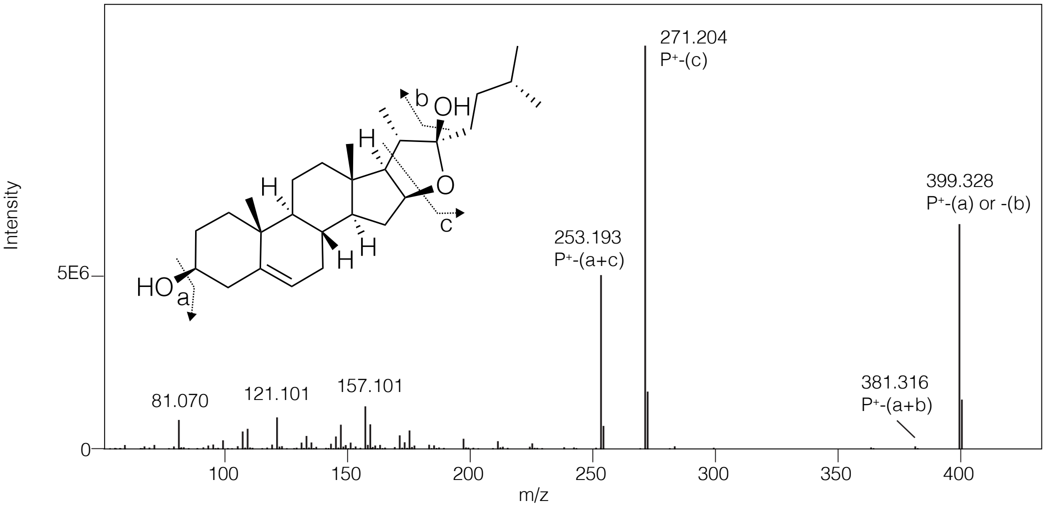

Supplementary Figure 12. MS[2] spectra of compounds 1-2. MS[2] fragmentation sites are shown. P^+, protonated precursor ion.

a

Compound 3, RT 18.5 min

M=$C_{27}H_{44}O_3$ / P^+=$C_{27}H_{45}O_3$ $[M+H]^+$

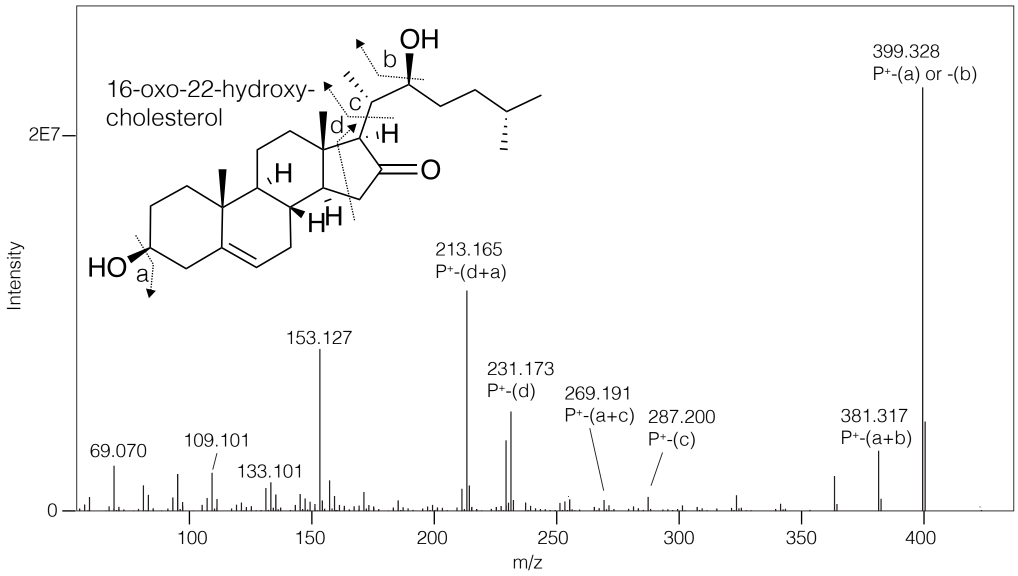

b

Compound 4, RT 17.8 min

M=$C_{27}H_{44}O_3$ / P^+=$C_{27}H_{45}O_3$ $[M+H]^+$

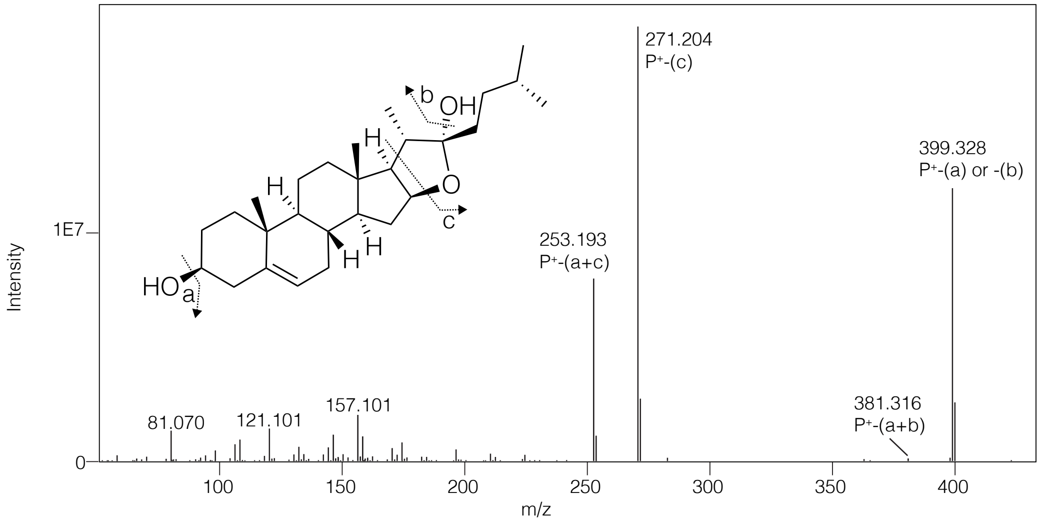

Supplementary Figure 13. MS^2 spectra of compounds 3-4 . MS^2 fragmentation sites are shown. P^+, protonated precursor ion.

a

Compound 5, RT 15.5 min

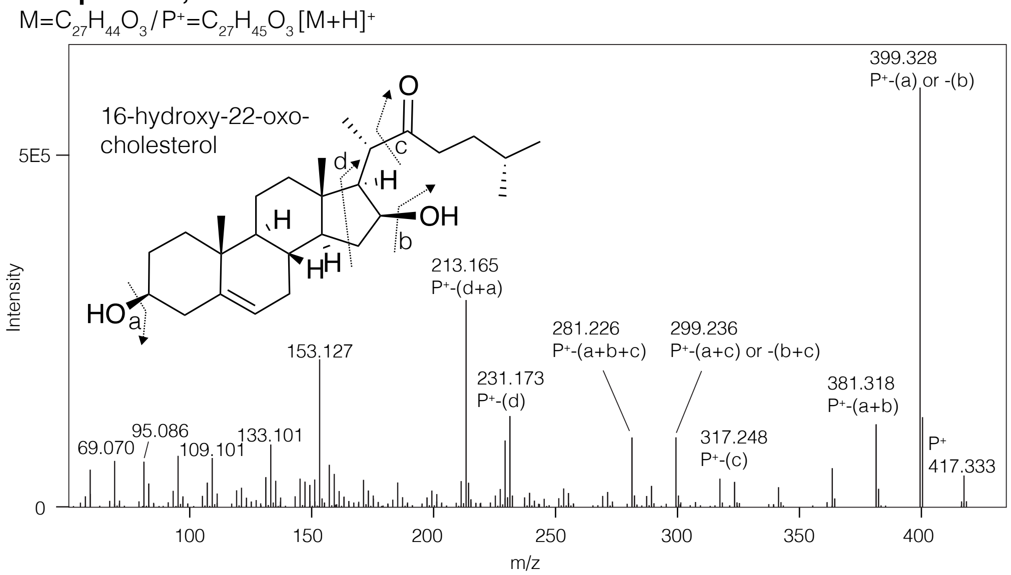

b

Diosgenin, RT 22.2 min

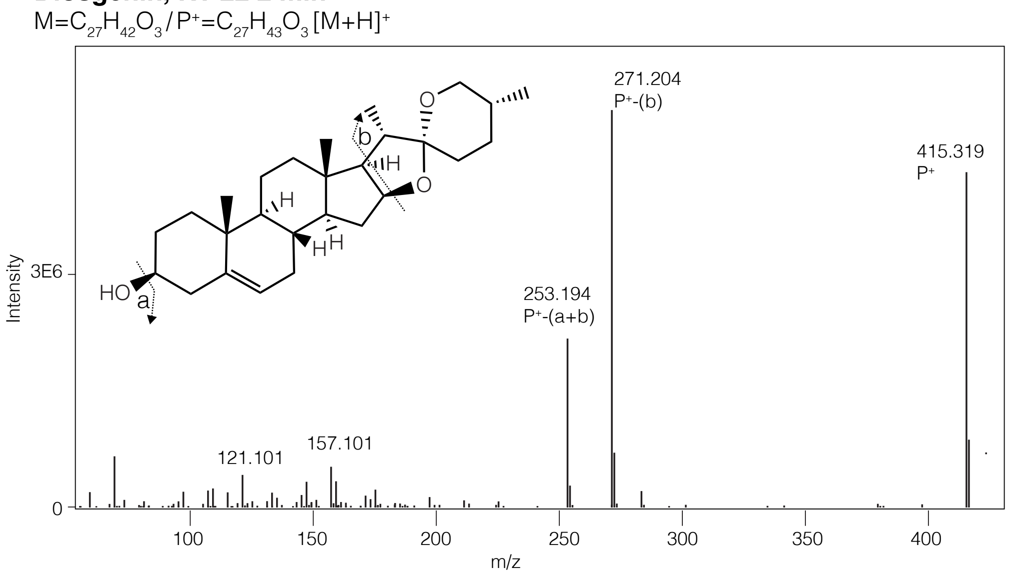

Supplementary Figure 14. MS^2 spectra of compounds 5 and diosgenin. MS^2 fragmentation sites are shown. P$^+$, protonated precursor ion.

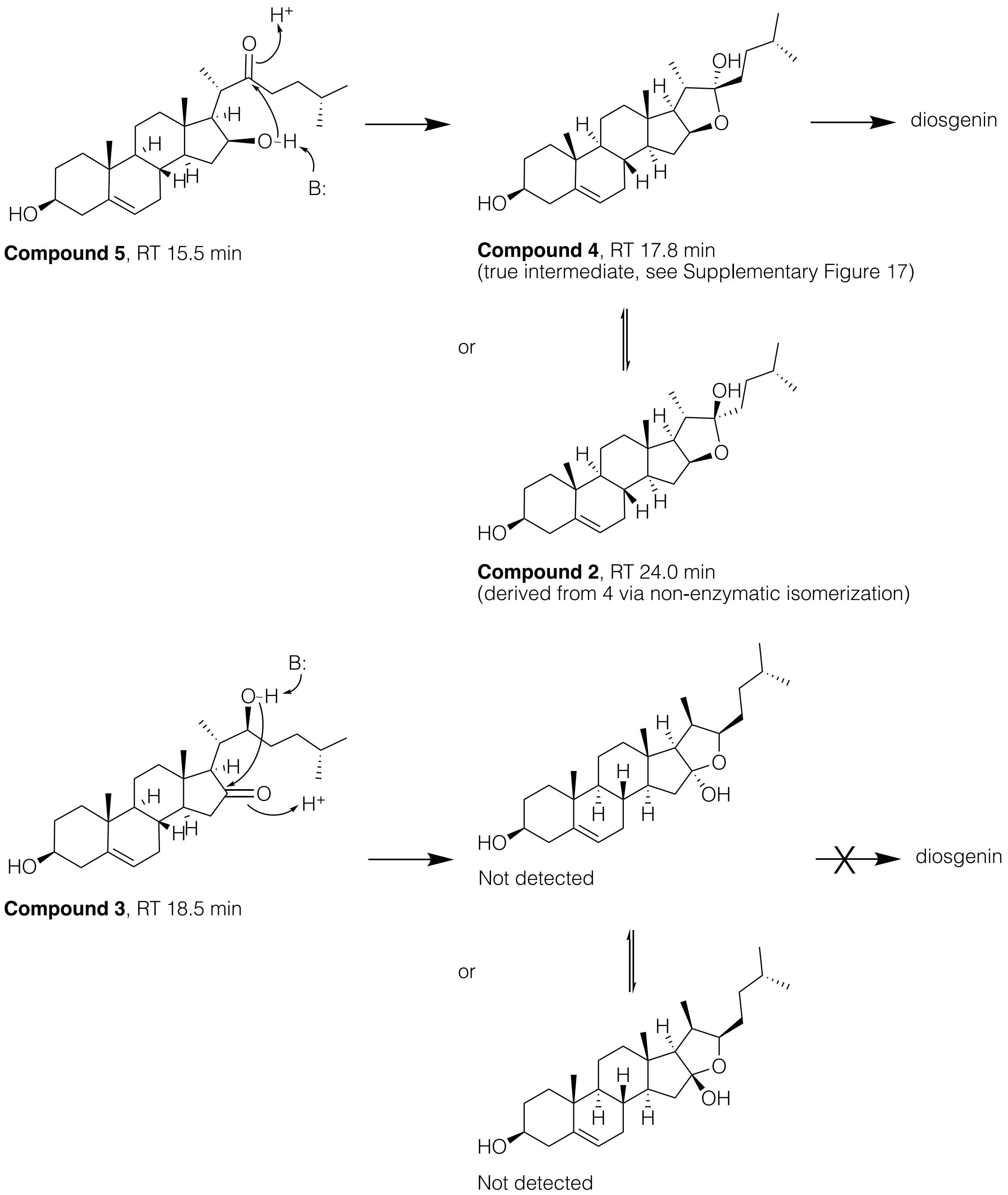

Supplementary Figure 15. Furoketalization of compound 5 and 3. Compounds 2 and 4 are two furostanol diastereomers resulted from furoketalization of compound 5. Products of furoketalization of compound 3 could not be detected but might be minor peaks observed in Figure 2d.

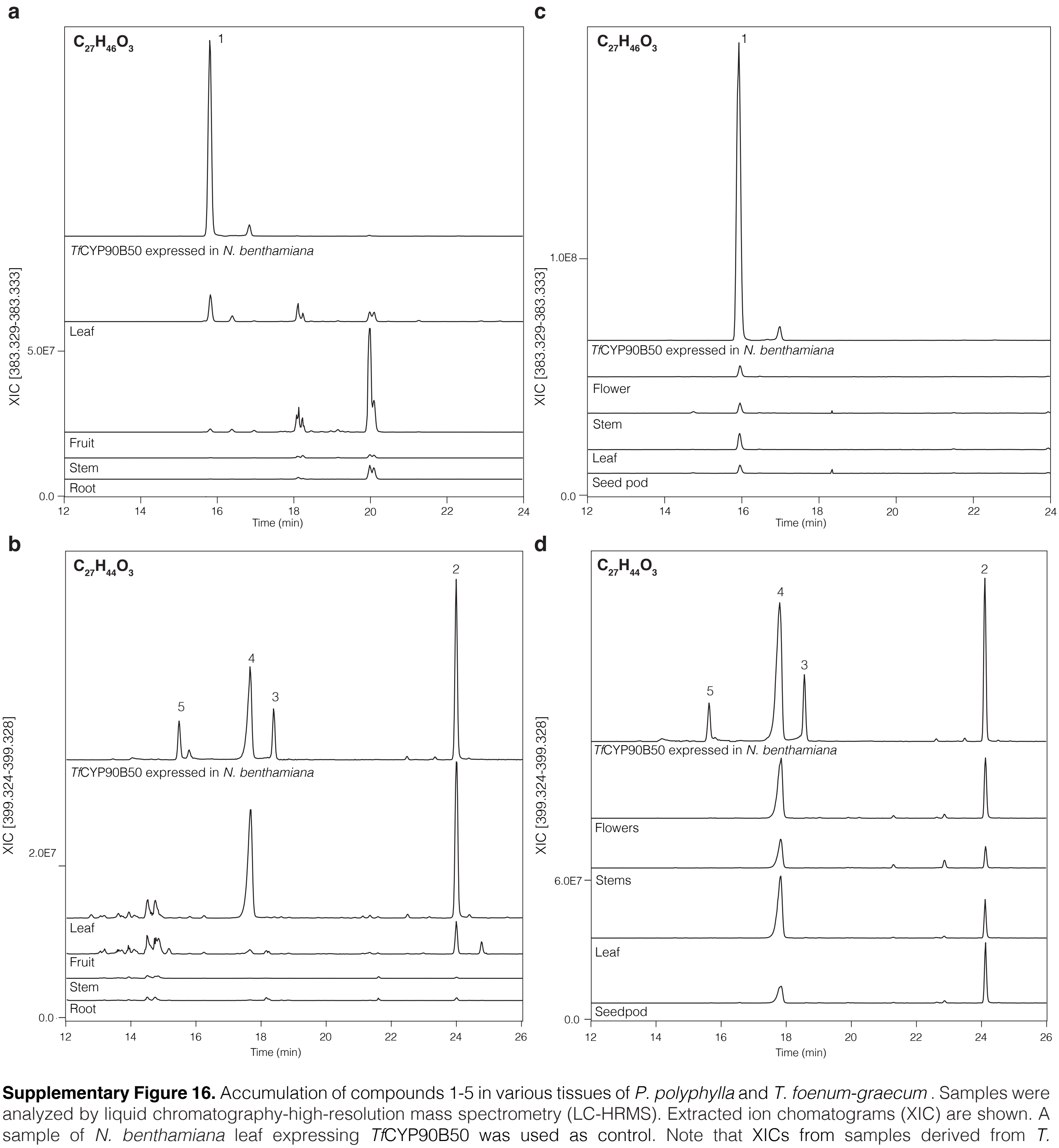

Supplementary Figure 16. Accumulation of compounds 1-5 in various tissues of *P. polyphylla* and *T. foenum-graecum* . Samples were analyzed by liquid chromatography-high-resolution mass spectrometry (LC-HRMS). Extracted ion chomatograms (XIC) are shown. A sample of *N. benthamiana* leaf expressing *Tf*CYP90B50 was used as control. Note that XICs from samples derived from *T. foenum-graecum* and *N. benthamiana* were aligned (retention time correction) because they were not analyzed within the same batch.

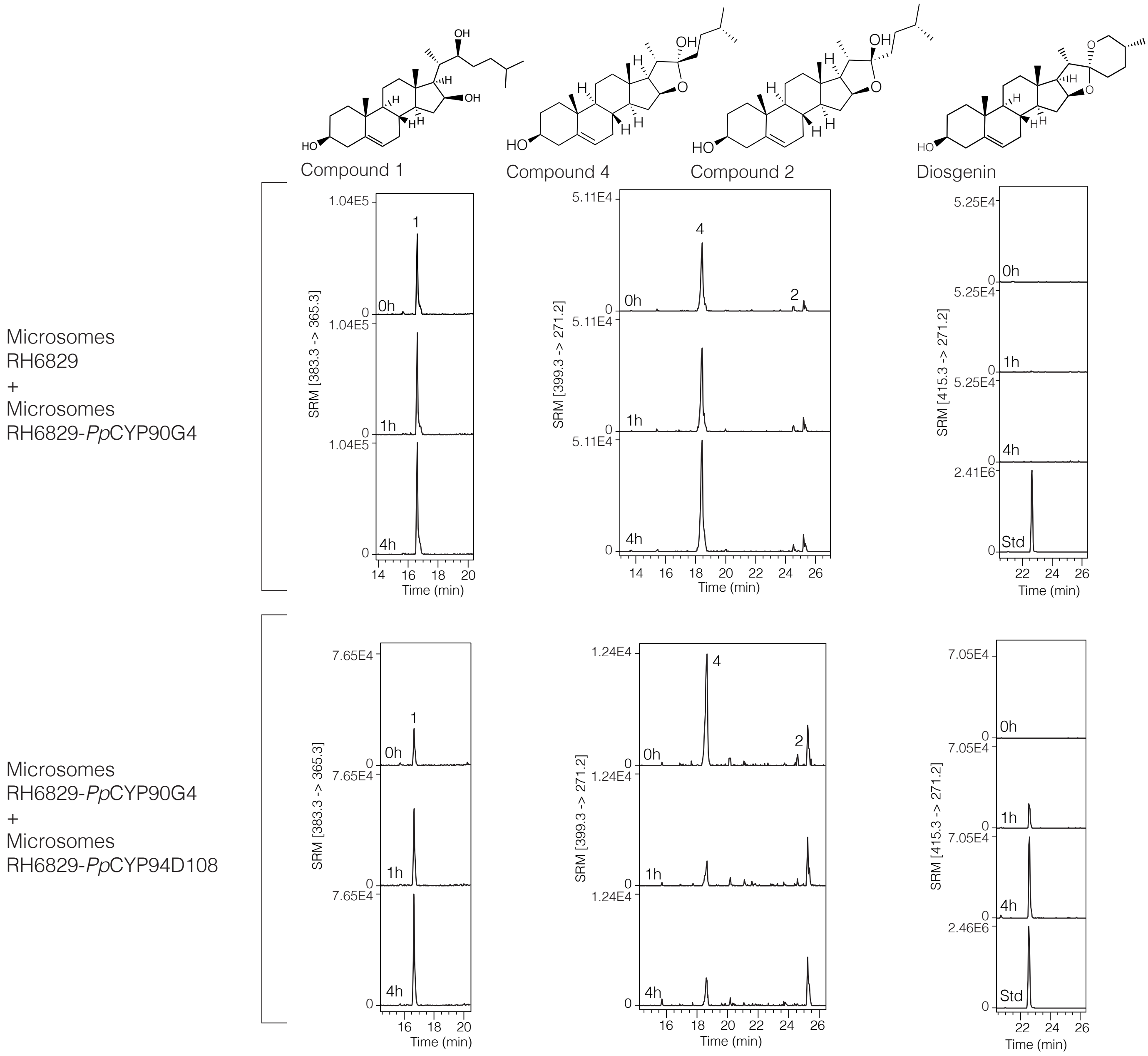

Supplementary Figure 17. In vitro enzyme assays using yeast microsomes. Microsomes were prepared from yeast strain RH6829 and RH6829 expressing either *Pp*CYP90G4 or *Pp*CYP94D108. Microsome preparations were combined as depicted and incubated at 30°C for 0, 1 and 4h. Assays were analyzed by liquid-chromatography mass-spectrometry (LC-MS). Pure diosgenin was used as standard. SRM, single reaction monitoring.

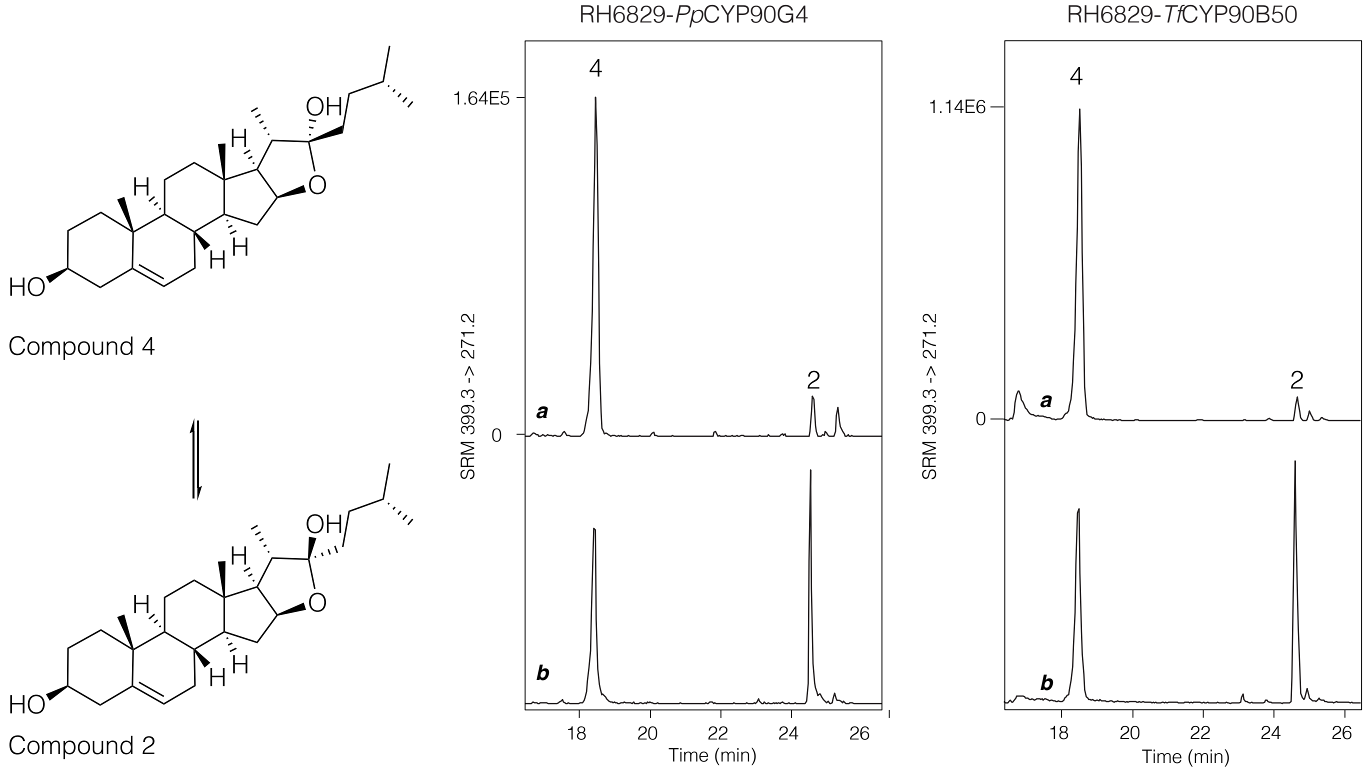

Supplementary Figure 18. Non-enzymatic isomerization of compound 2 to compound 4. Steroids extracted from yeast strain RH6829 expressing *Pp*CYP90G4 or *Tf*CYP90B50 were incubated at room temperature for a few days (***a***, before incubation; ***b***, after incubation) and then analyzed by liquid-chromatography mass-spectrometry (LC-MS). SRM, single reaction monitoring.

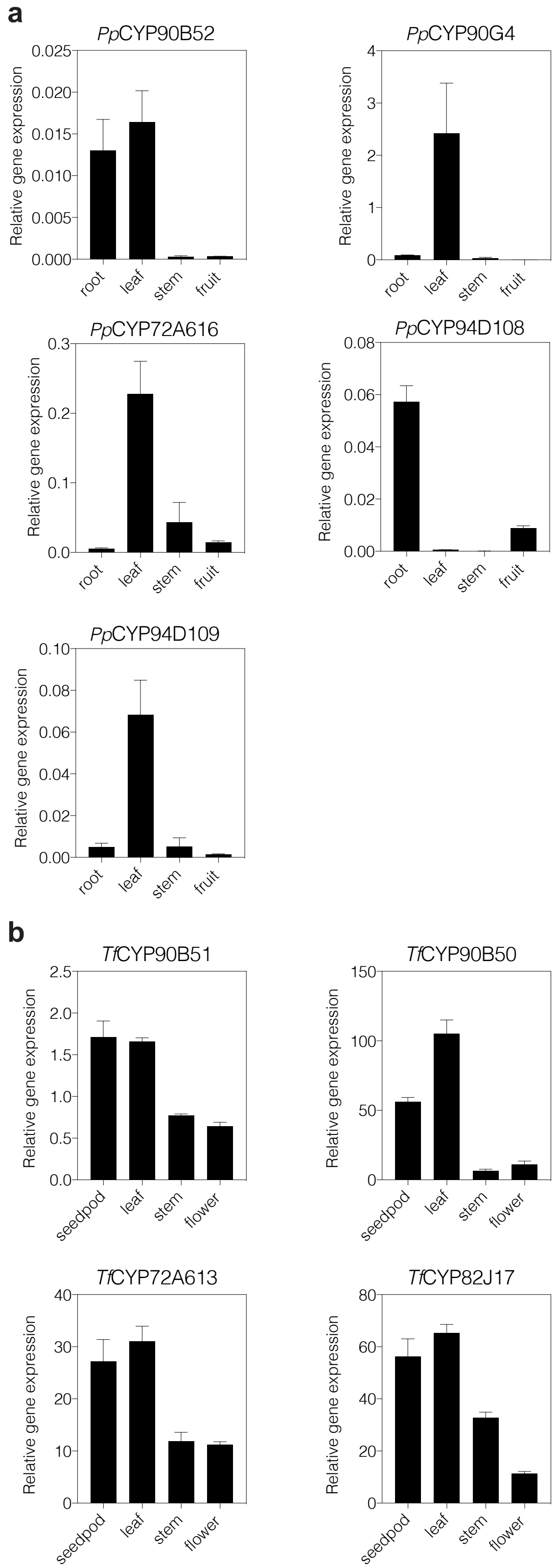

Supplementary Figure 19. Relative transcript levels of diosgenin-biosynthetic CYPs and BR-biosynthetic CYP90s. Gene expression was determined in various tissues of *P. polyphylla* and *T. foenum-graecum* by quantitative RT-PCR. Error bars, mean ± s.d. (n = 3 biological replicates). Gene expression values were calculated using Ct values and normalized using the following housekeeping genes: *P. polyphylla*, closest homolog to *Hordeum vulgare* alpha tubulin U40042.111; *T. foenum-graecum*, closest homolog to *Medicago truncatula* Medtr3g091400.112. Source data are provided as a Source Data file.

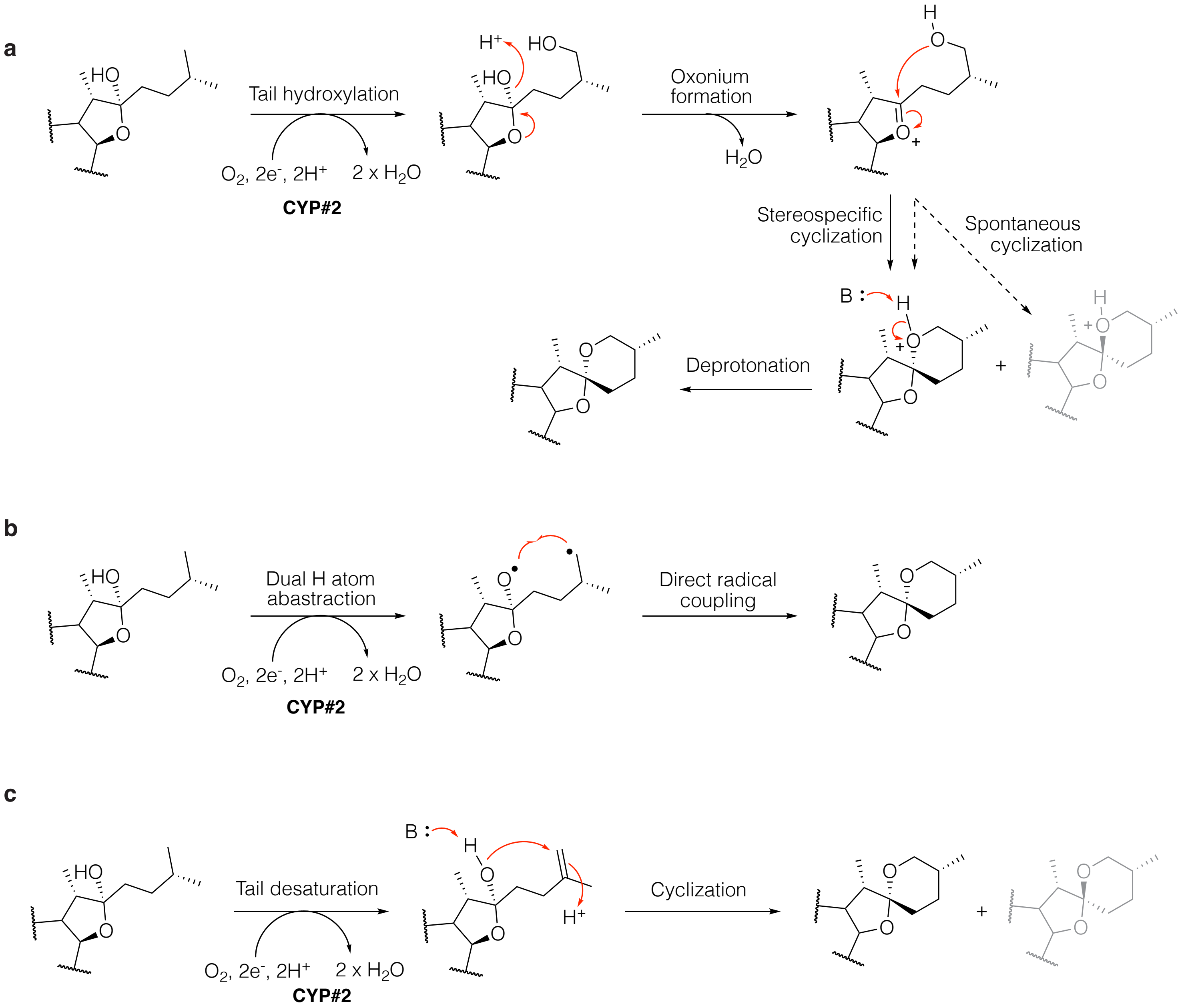

Supplementary Figure 20. Other possible mechanisms for the second cyclization step. (**a**) Dihydroxy-ketone cyclization mechanism analoguous to the one utilized by the bacterial cyclases AveC, MeiC and RevJ for 6,6-spiroketal production[4]. This mechanism can theoritically lead to the spontaneous formation of an enantiomeric mixture (chiral center at C22), but the CYP might guide a stereospecific cyclization. (**b**) Direct radical coupling cyclization mechanism resulting from dual H atom abstraction. (**c**) Cyclization mechanism triggered by tail desaturation. This mechanism can also theoritically lead to the spontaneous formation of an enantiomeric mixture (chiral center at C25).

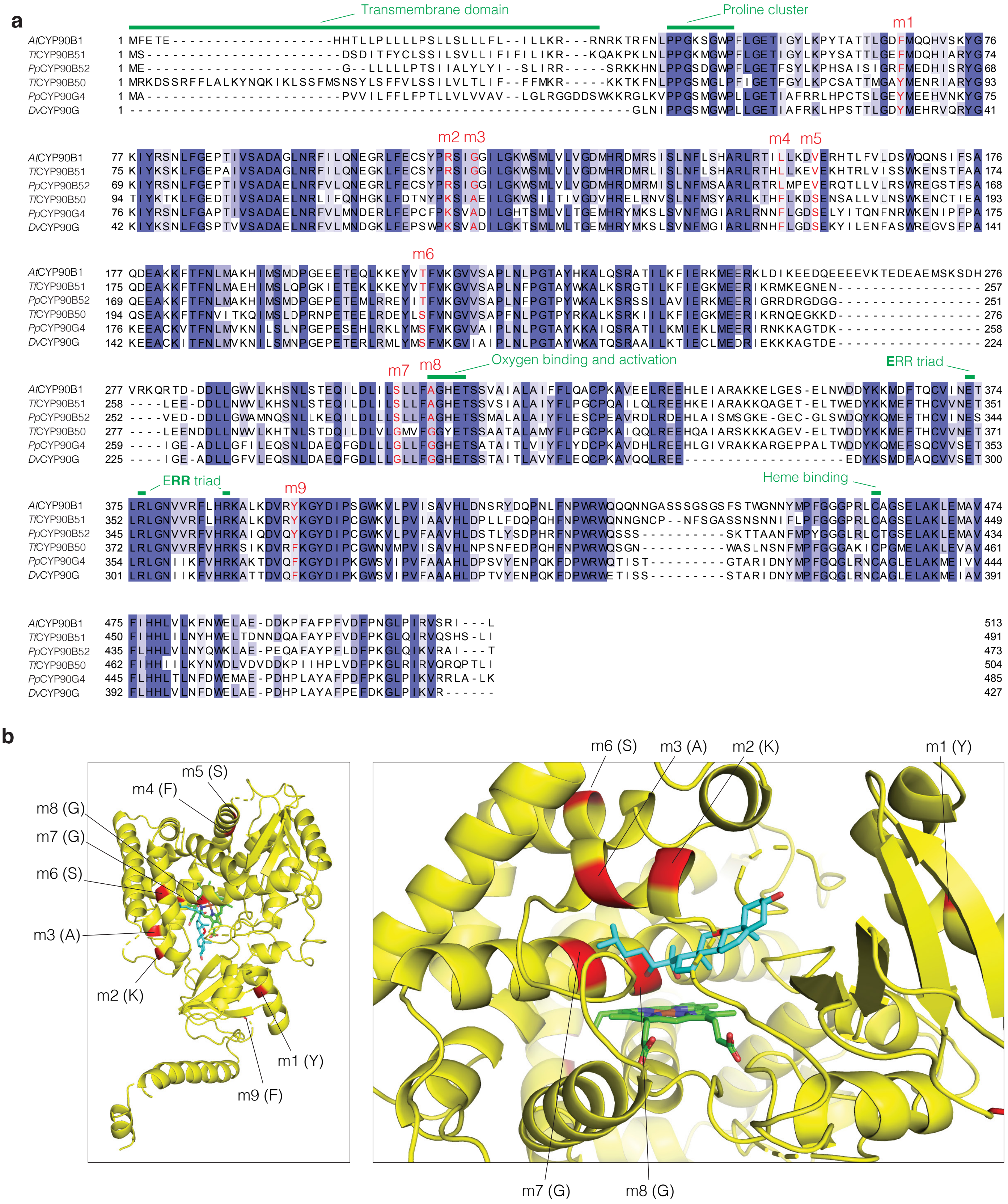

Supplementary Figure 21. Identification of residues differentially conserved between BR-biosynthetic and diosgenin-biosynthetic CYP90s. (**a**) Multiple sequence alignment of canonical BR-biosynthetic CYP90s and diosgenin-biosynthetic CYP90s. The nine residue positions that are differentially conserved between two groups of sequences are highlighted in red. The alignment was performed using Jalview V2 (T-Coffee, default settings[5]). See Fig. 5A for protein sequence accessions. (**b**) 3D protein model of diosgenin-biosynthetic *Tf*CYP90B50. The model was build using Phyre 2[6]. The nine residue positions that are differentially conserved between two groups of sequences are highlighted in red. *Tf*CYP90B50 was aligned in PyMOL (Schrödinger) with the crystal structure of human CYP11A1 in complex with 20,22-dihydroxycholesterol (PBD: 3NA0[7]), of which heme and 20,22-dihydroxycholesterol are shown.

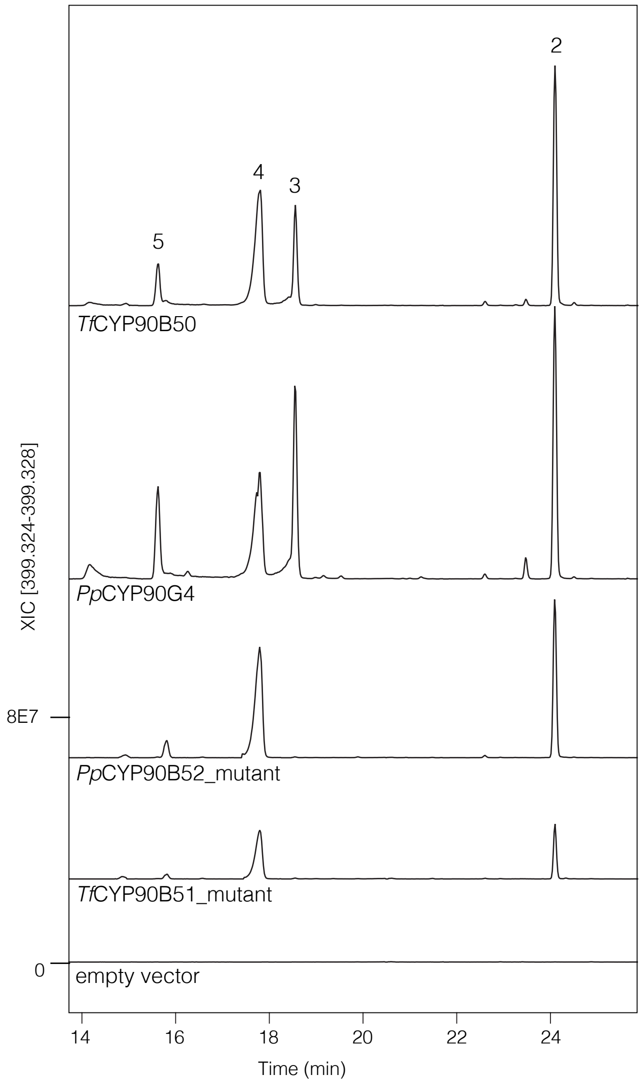

Supplementary Figure 22. Accumulation of compounds 2-5 in *N. benthamiana* expressing *Pp*CYP90B52_mutant and *Tf*CYP90B51_mutant. Samples were analyzed by liquid chromatography - high-resolution mass spectrometry (LC-HRMS). XIC, extracted ion chromatogram.

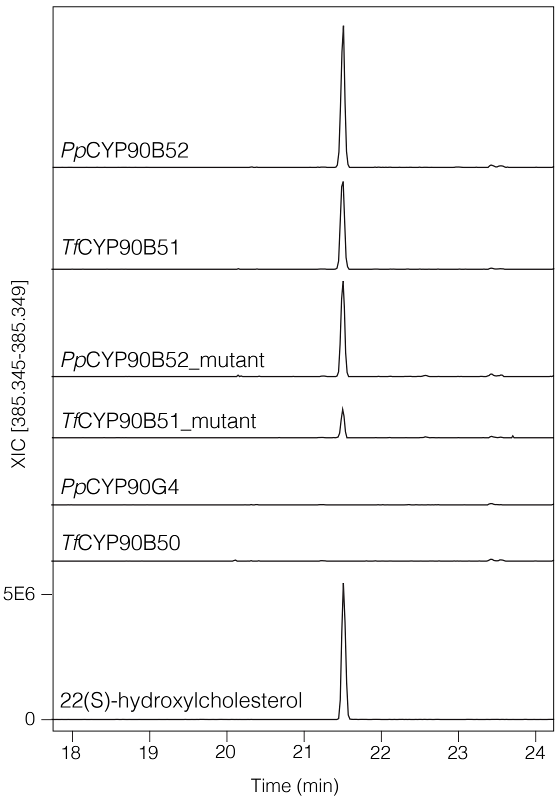

Supplementary Figure 23. Accumulation of 22(S)-hydroxycholesterol in *N. benthamiana* expressing *Pp*CYP90B52_mutant and *Tf*CYP90B51_mutant. Samples were analyzed by liquid chromatography - high-resolution mass spectrometry (LC-HRMS). XIC, extracted ion chromatogram. Pure 22(S)-hydroxylcholesterol was used as standard.

Supplementary Tables

Supplementary Table 1. Crystallographic parameters

Formula of asymmetric unit	$C_{243}H_{270}Cl_{24}N_{48}O_{18}Zn_{12}$
Molecular weight	5786.29
Crystal color, habit	Colorless, block
Crystal system	Monoclinic
a (Å)	31.0167(15)
b (Å)	14.3695(6)
c (Å)	31.6044(16)
β (°)	98.544(3)
V (Å^3)	13929.6(11)
Z	2
Density (g/cm^{-3})	1.380
Crystal size (μm^3)	221 × 72 × 41
Theta (*θ*) range for data collection	1.413° < *θ* < 74.390°
Linear absorption coefficient (mm^{-1})	3.748
Space group	$P2_1$
R_{int}	0.0905
R_1	0.1034
wR_2	0.3058
Number of parameters	3182
Number of restraints	976
Highest electron density maximum (eÅ^{-3})	2.85
Deepest electron density hole (eÅ^{-3})	–0.729
GoF	1.237
Flack parameter calculated by the Parsons' method[8]	0.120(5)
CCDC deposit number	1899808

Supplementary Table 2. ^{1}H and ^{13}C chemical shifts of compound 1

Carbon atom	^{1}H	^{13}C	Carbon atom	^{1}H	^{13}C
1	1.06 (1H, m)<br>1.86 (1H, m)	37.2	15	1.20 (1H, m)<br>2.25 (1H, m)	35.8
2	1.27 (2H, m)	29.9	16	4.35(1H, m)	72.0
3	3.53 (1H, m)	71.8	17	1.22 (1H, m)	57.2
4	2.28 (2H, m)	42.3	18	0.95 (3H, s)	13.0
5		121.6	19	1.03 (3H, s)	19.4
6	5.35 (1H, m)	140.8	20	2.30 (1H, m)	34.7
7	1.85 (2H, m)	31.6	21	0.99 (3H, d, *J*=7.2Hz)	16.3
8	1.51 (1H, m)	31.8	22	3.64 (1H, d, *J*=9.2Hz)	78.1
9	0.94 (1H, m)	50.1	23	1.58 (1H, m)	31.5
10		36.5	24	1.20 (1H, m)	36.1
11	1.50 (2H, m)	20.8	25	1.57 (1H, m)	28.2
12	1.14 (1H, m)<br>1.98 (1H, m)	40.0	26	0.93 (3H, d, *J*=3.2Hz)	22.5
13		42.5	27	0.92 (3H, d, *J*=3.2Hz)	22.8
14	0.92 (1H, m)	54.6			

Supplementary Table 3. Yeast strains used in this study

Strain name or Weng lab ID	Genes	Plasmid(s) transformed	Background strain	Genotype
RH6829	See[9]	See[9]	See[9]	MATa ura3 leu2 his3 trp1 can1 bar1 erg5D::HIS5-TDH3-DHCR24 erg6D::TRP1-TDH3-DHCR7
JKW-35.29	*Pp*CYP90G4, *At*ATR1	pJKW 1813 (stable integration)	RH6829	MATa ura3 leu2::LEU2-pGAL1-*Pp*CYP90G4-tTDH3-pGAL1-*Pp*CYP90G4-tTDH3-pCUP1-ATR1-pTDH3 his3 trp1 can1 bar1 erg5D::HIS5-TDH3-DHCR24 erg6D::TRP1-TDH3-DHCR7
JKW-34.24	*Pp*CYP94D108 *At*ATR1	pJKW 1847 (stable integration)	RH6829	MATa ura3::URA3-pGAL1-*Pp*CYP94D108-tTDH3-pGAL1-*Pp*CYP94D108-tTDH3-pCUP1-ATR1-pTDH3 leu2 his3 trp1 can1 bar1 erg5D::HIS5-TDH3-DHCR24 erg6D::TRP1-TDH3-DHCR7
JKW-34.25	*Pp*CYP90G4, *Pp*CYP94D108 *At*ATR1	pJKW 1813, pJKW 1847 (stable integrations)	RH6829	MATa ura3::URA3-pGAL1-*Pp*CYP94D108-tTDH3-pGAL1-*Pp*CYP94D108-tTDH3-pCUP1-ATR1-pTDH3 leu2::LEU2-pGAL1-*Pp*CYP90G4-tTDH3-pGAL1-*Pp*CYP90G4-tTDH3-pCUP1-ATR1-pTDH3 his3 trp1 can1 bar1 erg5D::HIS5-TDH3-DHCR24 erg6D::TRP1-TDH3-DHCR7
JKW-40.53	*Tf*CYP90B50 ,*Tf*CYP82J17	pJKW 2174	RH6829	MATa ura3 leu2 his3 trp1 can1 bar1 erg5D::HIS5-TDH3-DHCR24 erg6D::TRP1-TDH3-DHCR7
JKW-40.54	*Tf*CYP82J17, *Tf*CYP90B50	pJKW 2175	RH6829	MATa ura3 leu2 his3 trp1 can1 bar1 erg5D::HIS5-TDH3-DHCR24 erg6D::TRP1-TDH3-DHCR7
JKW-40.55	*Tf*CYP90B50	pJKW 2176	RH6829	MATa ura3 leu2 his3 trp1 can1 bar1 erg5D::HIS5-TDH3-DHCR24 erg6D::TRP1-TDH3-DHCR7

Supplementary References

1. Felsenstein, J. Confidence limits on phylogenies: an approach using the bootstrap. *Evolution* **39**, 783–791 (1985).
2. Zuckerkandl, E. & Pauling, L. Evolutionary divergence and convergence in proteins. *Evolving Genes and Proteins* 97–166 (1965). doi:10.1016/b978-1-4832-2734-4.50017-6
3. Tamura, K., Stecher, G., Peterson, D., Filipski, A. & Kumar, S. MEGA6: Molecular Evolutionary Genetics Analysis version 6.0. *Mol. Biol. Evol.* **30**, 2725–2729 (2013).
4. Tang, M.-C., Zou, Y., Watanabe, K., Walsh, C. T. & Tang, Y. Oxidative cyclization in natural product biosynthesis. *Chem. Rev.* **117**, 5226–5333 (2017).
5. Notredame, C., Higgins, D. G. & Heringa, J. T-Coffee: A novel method for fast and accurate multiple sequence alignment. *J. Mol. Biol.* **302**, 205–217 (2000).
6. Kelley, L. A., Mezulis, S., Yates, C. M., Wass, M. N. & Sternberg, M. J. E. The Phyre2 web portal for protein modeling, prediction and analysis. *Nat. Protoc.* **10**, 845–858 (2015).
7. Strushkevich, N. *et al.* Structural basis for pregnenolone biosynthesis by the mitochondrial monooxygenase system. *Proc. Natl. Acad. Sci. U. S. A.* **108**, 10139–10143 (2011).
8. Parsons, S., Flack, H. D. & Wagner, T. Use of intensity quotients and differences in absolute structure refinement. *Acta Crystallogr. B Struct. Sci. Cryst. Eng. Mater.* **69**, 249–259 (2013).
9. Souza, C. M. *et al.* A stable yeast strain efficiently producing cholesterol instead of ergosterol is functional for tryptophan uptake, but not weak organic acid resistance. *Metab. Eng.* **13**, 555–569 (2011).
