# Supplementary material for: Repeated evolution of cytochrome P450-mediated spiroketal steroid biosynthesis in plants
Source: Nat Commun. 2019 Jul 19;10:3206. doi: 10.1038/s41467-019-11286-7 (PMC6642093; doi:10.1038/s41467-019-11286-7)
Supplement: Supplementary file 2 — Description of Additional Supplementary Files [file 41467_2019_11286_MOESM2_ESM.pdf]

Description of Additional Supplementary Files

File name: Supplementary Data 1

Description: Plasmids generated in this study

File name: Supplementary Data 2

Description: CYPs selected for the screen presented in Figure 1

File name: Supplementary Data 3

Description: Primers used in this study
