# Supplementary material for: Repeated evolution of cytochrome P450-mediated spiroketal steroid biosynthesis in plants
Source: Nat Commun. 2019 Jul 19;10:3206. doi: 10.1038/s41467-019-11286-7 (PMC6642093; doi:10.1038/s41467-019-11286-7)
Supplement: Supplementary file 6 — Reporting Summary [file 41467_2019_11286_MOESM6_ESM.pdf]

nature research

Corresponding author(s): Jing-Ke Weng

Last updated by author(s): Jun 22, 2019

Reporting Summary

Nature Research wishes to improve the reproducibility of the work that we publish. This form provides structure for consistency and transparency in reporting. For further information on Nature Research policies, see Authors & Referees and the Editorial Policy Checklist.

Statistics

For all statistical analyses, confirm that the following items are present in the figure legend, table legend, main text, or Methods section.

n/a	Confirmed	
☐	☒	The exact sample size (*n*) for each experimental group/condition, given as a discrete number and unit of measurement
☐	☒	A statement on whether measurements were taken from distinct samples or whether the same sample was measured repeatedly
☒	☐	The statistical test(s) used AND whether they are one- or two-sided<br>*Only common tests should be described solely by name; describe more complex techniques in the Methods section.*
☒	☐	A description of all covariates tested
☒	☐	A description of any assumptions or corrections, such as tests of normality and adjustment for multiple comparisons
☒	☐	A full description of the statistical parameters including central tendency (e.g. means) or other basic estimates (e.g. regression coefficient) AND variation (e.g. standard deviation) or associated estimates of uncertainty (e.g. confidence intervals)
☒	☐	For null hypothesis testing, the test statistic (e.g. *F*, *t*, *r*) with confidence intervals, effect sizes, degrees of freedom and *P* value noted<br>*Give P values as exact values whenever suitable.*
☒	☐	For Bayesian analysis, information on the choice of priors and Markov chain Monte Carlo settings
☒	☐	For hierarchical and complex designs, identification of the appropriate level for tests and full reporting of outcomes
☒	☐	Estimates of effect sizes (e.g. Cohen's *d*, Pearson's *r*), indicating how they were calculated

Our web collection on statistics for biologists contains articles on many of the points above.

Software and code

Policy information about availability of computer code

Data collection	LC-MS software used to collect data including Chromeleon and TSQ Tune (Thermo Fisher Scientific)<br>NMR software: TopSpin (Bruker)<br>X-ray software: SAINT and SADABS programs (Bruker).<br>qPCR data were collected on a QuantStudio(TM) 6 Flex System
Data analysis	Xcalibur Qual Browser (Thermo Fisher Scientific) and mzMINE 2.30-2.37 were used to analyze and extract LC-MS data.<br>NMR software: TopSpin (Bruker)<br>De novo transcriptome assembly: raw sequences were assembled using Trinity (v2.4.0) in strand-specific mode. Gene expression measured by transcripts per million (TPM) was calculated using RSEM (v1.3.0). Candidate coding regions within transcript sequences were identified using Transdecoder (v4.1.0) and annotation of the transcripts/predicted protein sequences was performed using in-house scripts. To extract full-length CYP candidates from Trinity-assembled transcriptomes, we used an in-house Python script (available on demand) that searches for transcripts containing the Pfam PF00067, having a length between 400 and 600 amino acids and having a STOP codon.<br>Raw data obtained using the crystalline sponge (CS) method were processed with the SAINT and SADABS programs (Bruker). All crystal structures were solved using SHELXT (ver. 2018/2)17 and refined using SHELXL (ver. 2018/3)<br>Graphs were built using GraphPad Prism (ver. 7.0).<br>Figures were compiled using Adobe Illustrator CC 2017.1.0.

For manuscripts utilizing custom algorithms or software that are central to the research but not yet described in published literature, software must be made available to editors/reviewers. We strongly encourage code deposition in a community repository (e.g. GitHub). See the Nature Research guidelines for submitting code & software for further information.

Data

Policy information about availability of data

All manuscripts must include a data availability statement. This statement should provide the following information, where applicable:

- Accession codes, unique identifiers, or web links for publicly available datasets
- A list of figures that have associated raw data
- A description of any restrictions on data availability

Provide your data availability statement here.

Field-specific reporting

Please select the one below that is the best fit for your research. If you are not sure, read the appropriate sections before making your selection.

☒ Life sciences ☐ Behavioural & social sciences ☐ Ecological, evolutionary & environmental sciences

For a reference copy of the document with all sections, see nature.com/documents/nr-reporting-summary-flat.pdf

Life sciences study design

All studies must disclose on these points even when the disclosure is negative.

Sample size	Std sample sizes in our field were chosen.
Data exclusions	No data was excluded.
Replication	Replications were all biological replicates.
Randomization	Samples were always analyzed by LC-MS in a random manner
Blinding	No blinding

Reporting for specific materials, systems and methods

We require information from authors about some types of materials, experimental systems and methods used in many studies. Here, indicate whether each material, system or method listed is relevant to your study. If you are not sure if a list item applies to your research, read the appropriate section before selecting a response.

Materials & experimental systems

n/a	Involved in the study	
☒	☐	Antibodies
☒	☐	Eukaryotic cell lines
☒	☐	Palaeontology
☒	☐	Animals and other organisms
☒	☐	Human research participants
☒	☐	Clinical data

Methods

n/a	Involved in the study	
☒	☐	ChIP-seq
☒	☐	Flow cytometry
☒	☐	MRI-based neuroimaging
